# The cholesterol biosynthesis enzyme FAXDC2 couples Wnt/**β**-catenin to RTK/MAPK signaling

**DOI:** 10.1172/JCI171222

**Published:** 2024-01-23

**Authors:** Babita Madan, Shawn R. Wadia, Siddhi Patnaik, Nathan Harmston, Emile Tan, Iain Bee Huat Tan, W. David Nes, Enrico Petretto, David M. Virshup

**Affiliations:** 1 Program in Cancer and Stem Cell Biology, Duke-NUS Medical School, Singapore.; 2 Science Division, Yale-NUS College, Singapore.; 3 Department of Colorectal Surgery, Singapore General Hospital, Singapore.; 4 Department of Medical Oncology, National Cancer Centre, Singapore.; 5 Department of Chemistry and Biochemistry, Texas Tech University, Lubbock, Texas, USA.; 6 Center for Computational Biology and Program in Cardiovascular and Metabolic Disorders, Duke-NUS Medical School, Singapore.; 7 Institute for Big Data and Artificial Intelligence in Medicine, School of Science, China Pharmaceutical University, Nanjing, China.; 8 Department of Pediatrics, Duke University School of Medicine, Durham, North Carolina, USA.

**Keywords:** Metabolism, Oncology, Cancer, Cholesterol, Phosphotyrosine

## Abstract

Wnts, cholesterol, and MAPK signaling are essential for development and adult homeostasis. Here, we report that fatty acid hydroxylase domain containing 2 (FAXDC2), a previously uncharacterized enzyme, functions as a methyl sterol oxidase catalyzing C4 demethylation in the Kandutsch-Russell branch of the cholesterol biosynthesis pathway. FAXDC2, a paralog of MSMO1, regulated the abundance of the specific C4-methyl sterols lophenol and dihydro-T-MAS. Highlighting its clinical relevance, FAXDC2 was repressed in Wnt/β-catenin–high cancer xenografts, in a mouse genetic model of Wnt activation, and in human colorectal cancers. Moreover, in primary human colorectal cancers, the sterol lophenol, regulated by FAXDC2, accumulated in the cancerous tissues and not in adjacent normal tissues. FAXDC2 linked Wnts to RTK/MAPK signaling. Wnt inhibition drove increased recycling of RTKs and activation of the MAPK pathway, and this required FAXDC2. Blocking Wnt signaling in Wnt-high cancers caused both differentiation and senescence; and this was prevented by knockout of FAXDC2. Our data show the integration of 3 ancient pathways, Wnts, cholesterol synthesis, and RTK/MAPK signaling, in cellular proliferation and differentiation.

## Abstract 2

FAXDC2 is a Wnt-repressed cholesterol biosynthesis enzyme that regulates the abundance of C4-methyl sterol, lophenol, and RTK/MAPK signaling in cancers.

## Graphical abstract

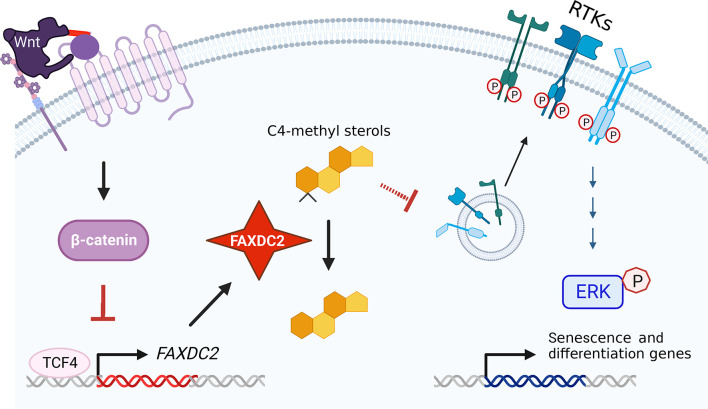

## Introduction

During animal development and in adult stem cell niches, the balance between stemness, proliferation, and differentiation is regulated by Wnt/β-catenin signaling (1, 2). Wnts are secreted palmitoleated glycoproteins that bind to their cognate receptors on nearby cells to influence multiple downstream signaling pathways in a context-dependent manner. When Wnt signaling is defective, developmental anomalies occur. When Wnt signaling is hyperactive, stem cell differentiation is impaired, and this predisposes to malignancy (3). Studying the pathways regulated by Wnts can reveal both the cellular signaling networks that mediate essential cell fate decisions and the mechanisms of cancer development.

Mutations that cause an accumulation of Wnt receptors on the cell surface (e.g., loss of RNF43 or overexpression of RSPO3) (4–6) lead to hypersensitivity and addiction to Wnts. These mutations enhance Wnt signaling and drive the development of diverse cancers (7). Porcupine (PORCN) is an *O*-acyltransferase that adds a palmitoleate moiety to all 19 Wnts at a conserved serine residue, an essential step in Wnt ligand secretion (8–10). Pharmacologic targeting of PORCN blocks Wnt secretion and prevents the growth of Wnt ligand–dependent cancers. The treatment of Wnt ligand–dependent preclinical xenograft models with drugs that inhibit Wnt palmitoleation provides a powerful system for studying the pathways regulated by Wnt signaling (4, 11–13).

To identify Wnt-regulated genes and pathways, we examined the transcriptional response of multiple Wnt ligand–addicted cancer xenografts following PORCN inhibition (14). Our studies confirmed broad control of transcription by Wnt signaling. In these xenograft models, roughly a third of expressed genes were Wnt activated, and another third were Wnt repressed; that is, they had increased expression upon Wnt inhibition. Orthotopic xenograft tumor models identified significantly more Wnt-regulated genes than are found in tissue culture systems (14, 15). This is likely due to the important contribution of the local microenvironment that provides ligands and interactions that are not found in cell culture. For example, these in vivo studies have identified ribosomal biogenesis and homologous recombination pathways as being Wnt activated. Recently we also demonstrated that, counterintuitively, Wnt signaling inhibits mitogen-activated protein kinase (MAPK) signaling, a pathway critical for both proliferation and differentiation (16, 17).

Cholesterol biosynthesis is another ancient pathway required for growth. Rapidly proliferating tissues require large amounts of cholesterol for new cell membranes. In addition, cholesterol biosynthesis provides precursors to produce diverse signaling sterols including vitamin D, bile acids, corticosteroids, and sex hormones. Cellular cholesterol is obtained through both diet and de novo synthesis. The synthesis of cholesterol from lanosterol proceeds via 2 parallel pathways, the Bloch and Kandutsch-Russell (KR) pathways (18–20). Changes in the flux through these pathways alter the abundance of specific cholesterol biosynthesis intermediates, several of which have known biological signaling activity. For example, the C4-dimethyl sterols testicular meiosis-activating sterol (T-MAS) and follicular fluid meiosis-activating sterol (FF-MAS) regulate germ cell development and EGFR signaling in cancer cells, and the C4-monomethyl sterol lophenol regulates cell fate decisions in *Caenorhabditis* *elegans* (21–23). Individuals with mutations in the genes catalyzing the oxidative removal of C4-methyl groups accumulate these sterols in affected tissues, leading to anomalies such as microcephaly, bilateral congenital cataracts, growth delay, psoriasiform dermatitis, immune dysfunction, and intellectual disability (24–27). Thus, modulation of cholesterol signaling intermediates is critical in development, but whether this is regulated by Wnt signaling is not known.

Here, exploiting potent inhibitors of Wnt production and powerful in vivo models, we describe the interaction of Wnt signaling with cholesterol biosynthesis, leading to the regulation of RTK/MAPK signaling. We identify the cholesterol biosynthesis enzyme fatty acid hydroxylase domain containing 2 (FAXDC2) as a key control point. This study establishes the interaction of 3 ancient and core developmental pathways via cholesterol precursor metabolites, coupling Wnt and RTK/MAPK signaling with broad implications for both normal and cancer biology.

## Results

### Wnt signaling represses the cholesterol biosynthesis enzyme FAXDC2.

**Figure 1:**
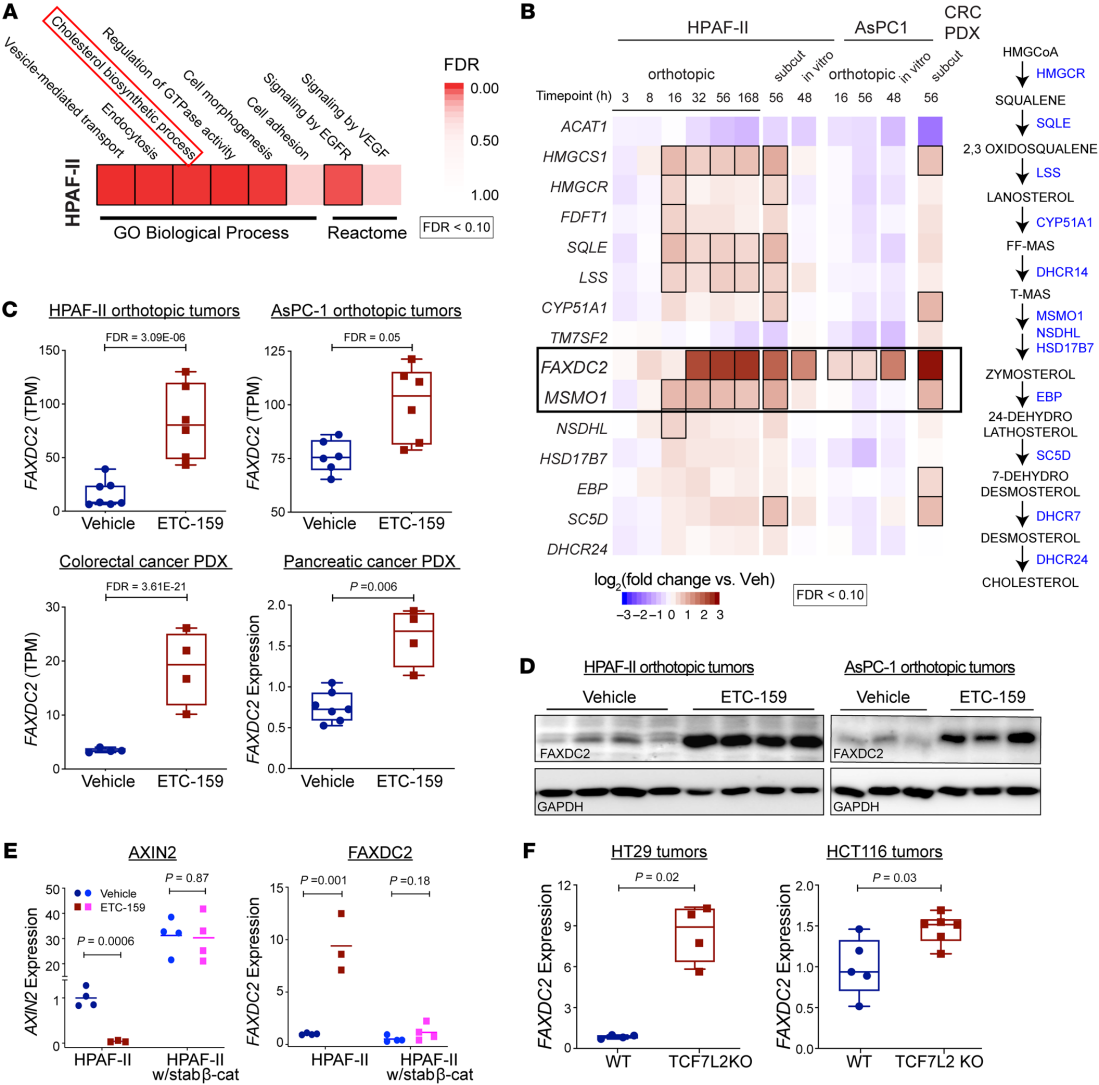
Wnt signaling represses the cholesterol biosynthesis enzyme FAXDC2. (**A**) Wnt inhibition upregulates genes regulating cholesterol biosynthesis. Pharmacologic Wnt inhibition with ETC-159 in HPAF-II orthotopic xenografts upregulates 4,350 genes that are grouped into temporal clusters (*n* = 4–6 mice per group). GO Biological Process and Reactome analysis of the *Wnt*-repressed genes highlights processes including cholesterol biosynthesis, vesicle-mediated transport, and EGFR/VEGF signaling (hypergeometric test, FDR < 10%). (**B**) Wnt signaling represses genes encoding enzymes in the cholesterol biosynthesis pathway. Left: Wnt inhibition with ETC-159 increases expression of multiple cholesterol biosynthesis pathway genes in 3 independent systems: HPAF-II and AsPC-1 orthotopic xenografts and cells in culture and colorectal PDX (14) (*n* = 4–6 tumors per group). Boxed genes show significant change in expression (FDR < 0.10). Right: Cholesterol biosynthesis pathway. (**C**) Wnt inhibition increases FAXDC2 expression in multiple Wnt-addicted cancer models: ETC-159–treated HPAF-II, AsPC-1 orthotopic tumors, and Wnt-addicted colorectal and pancreatic cancer PDX models have 2- to 10-fold higher *FAXDC2* expression compared with control tumors. Relative expression from RNA-Seq in TPM or quantitative reverse transcription PCR (qRT-PCR) is shown. Each data point represents an independent tumor, *n* = 5–6 per group (hypergeometric test, FDR < 10%, or Mann-Whitney test). (**D**) ETC-159 treatment increases FAXDC2 protein abundance in tumors. Protein lysates from HPAF-II and AsPC-1 orthotopic xenografts from vehicle- or ETC-159–treated mice were probed with FAXDC2 or GAPDH antibodies. Each lane represents tumor lysate from an individual mouse. (**E**) Stabilized β‑catenin suppresses FAXDC2 expression despite upstream Wnt inhibition by ETC-159. Mice bearing xenografts from control HPAF-II cells or cells expressing stabilized β‑catenin were treated with ETC-159 or vehicle for 56 hours. *FAXDC2* and *AXIN2* mRNA were quantitated by qRT-PCR and normalized to both *ACTB* and *EPN1*. *AXIN2* upregulation is a control for stabilized β‑catenin activity. (**F**) Genetic inhibition of Wnt signaling by TCF7L2 knockout in HT29 and HCT116 colon cancer xenografts increases FAXDC2 expression 2- to 8-fold in comparison with WT controls. (**E** and **F**) Each data point represents an independent tumor. Unpaired *t* test was used to calculate *P* values.

To identify Wnt-regulated genes in cancers, we previously performed a comprehensive transcriptional analysis of multiple Wnt ligand–dependent models driven by distinct genetic mutations (11, 14, 16). These studies revealed that out of 11,673 expressed genes, inhibiting Wnt signaling decreased the expression of 3,549 (30%) and increased the expression of 4,350 (37%) genes (Supplemental Figure 1A; supplemental material available online with this article; https://doi.org/10.1172/JCI171222DS1). One unexpected finding was that Wnt signaling repressed the expression of multiple genes involved in cholesterol biosynthesis (Figure 1, A and B).

The gene in the cholesterol biosynthesis pathway with the most significant and general upregulation after inhibition of Wnt signaling was fatty acid hydroxylase domain containing 2 (*FAXDC2*; C5orf4) (Figure 1B), a putative paralog of methyl sterol monooxygenase 1 (MSMO1) about which little is known. Treatment of mice bearing HPAF-II orthotopic tumors with a pan-Wnt secretion inhibitor, ETC-159, led to a robust upregulation of *FAXDC2* mRNA (7.5-fold after 2 days of treatment) (Figure 1, B and C). *FAXDC2* expression was also markedly enhanced by Wnt inhibition in 4 independent Wnt-high cancer models driven by distinct genetic mutations. These included AsPC-1 pancreatic cancer orthotopic xenografts and pancreatic cancer patient-derived xenograft (PDX) PAXF1861, both with loss-of-function RNF43 mutations, and a colorectal PDX with an R-spondin translocation (Figure 1, B and C). This confirms that regulation of *FAXDC2* by Wnts is widespread. Consistent with the change in gene expression, we found a marked increase in FAXDC2 protein abundance in both Wnt-inhibited HPAF-II and AsPC-1 orthotopic tumors (Figure 1D).

Wnt pathway inhibition causes phosphorylation-mediated degradation of β-catenin. To test whether degradation of β-catenin is required for the robust upregulation of *FAXDC2* and other genes in the cholesterol biosynthesis pathway, we generated HPAF-II cells that express stabilized β-catenin (16). β-Catenin with mutated serine residues (S33A, S37A, T41A, and S45A) is insensitive to CK1α/GSK3 phosphorylation, preventing its proteasomal degradation. HPAF-II tumors expressing stabilized β-catenin have sustained downstream activation of the Wnt/β-catenin pathway regardless of PORCN inhibition, as evidenced by the failure of PORCN inhibition to reduce the expression of the Wnt target gene *AXIN2* (Figure 1E, left). Stabilized β-catenin prevented the upregulation of several cholesterol pathway genes. Notably, the gene with the highest dynamic range was *FAXDC2* (Figure 1E and Supplemental Figure 1B). Thus, active β-catenin represses *FAXDC2* expression.

To complement the PORCN inhibitor assays, we performed a genetic test of the role of β-catenin signaling in suppressing *FAXDC2* expression. We examined the impact of knocking out β-catenin’s nuclear binding partner, TCF4, which is encoded by the *TCF7L2* gene (28). APC-mutant HT29 and β-catenin–mutant HCT116 colorectal cancer cells with or without *TCF7L2* knockout were obtained from the Hecht group (Albert Ludwigs University of Freiburg), and xenograft tumors were generated (29). *TCF7L2*-KO tumors had, as previously demonstrated, decreased β-catenin signaling as evidenced by reduced *AXIN2* expression (Supplemental Figure 1, C and D). Notably, both HT29 *TCF7L2-*KO and HCT116 *TCF7L2-*KO xenograft tumors showed approximately 2- to 10-fold increase in the expression of *FAXDC2* (Figure 1F). These data establish that the Wnt/β-catenin/TCF4 axis strongly represses *FAXDC2* expression.

SREBP1 and SREBP2 are well-established regulators of several genes in the cholesterol biosynthesis pathway. To directly test the impact of SREBP1 and SREBP2 on *FAXDC2* expression, we used multiple siRNAs to knock down these genes in HPAF-II cells. We find that knockdown of neither SREBP1 nor SREBP2 affected Wnt inhibition–induced increase in *FAXDC2* expression (Supplemental Figure 1, E and F). We conclude that SREBP regulates neither basal nor Wnt-regulated FAXDC2 expression.

### FAXDC2 is a C4-methyl sterol oxidase in the Kandutsch-Russell branch of the cholesterol biosynthesis pathway.

**Figure 2:**
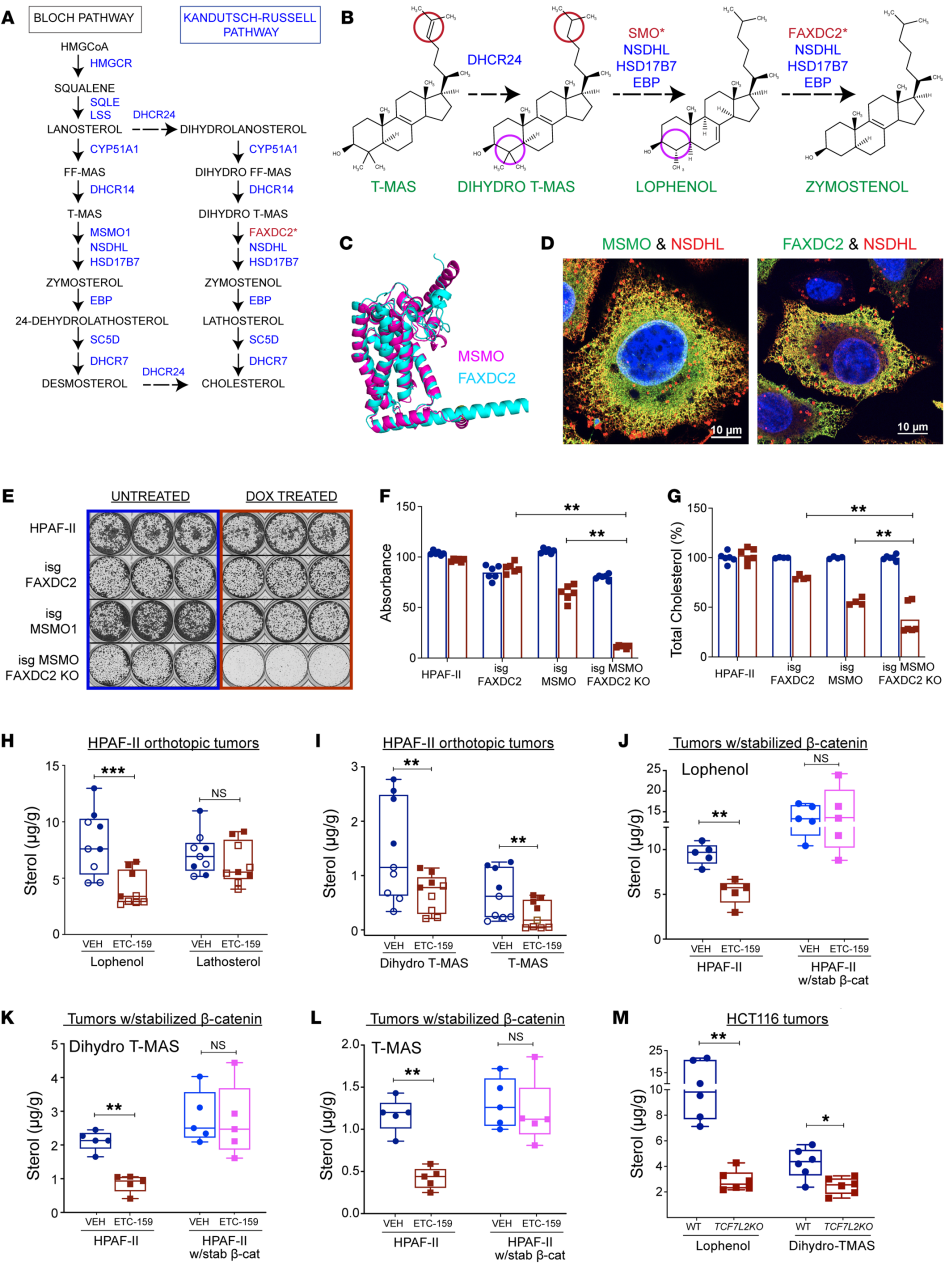
FAXDC2 is a C4-demethylase in the KR branch of the cholesterol biosynthesis pathway. (**A**) Postlanosterol cholesterol biosynthesis via Bloch and KR pathways, highlighting key steps and associated enzymes. *Proposed location of FAXDC2 in the pathway. (**B**) Conversion of T-MAS to zymostenol, highlighting reduction at C24 and demethylation at C4 involving sterol methyl oxidase (SMO). *SMO indicates either MSMO1 or FAXDC2 sterol methyl oxidase. (**C**) MSMO1 and FAXDC2 structures predicted by AlphaFold show considerable homology (root-mean-square deviation of atomic positions 1.36 Å). (**D**) Like MSMO1, FAXDC2 colocalizes with NSDHL in the SER. Epitope-tagged MSMO1, NSDHL, and FAXDC2 constructs were expressed in HeLa cells, and their localization was visualized by staining with fluorescence-tagged anti-epitope antibodies. (**E**–**G**) Combined knockdown of FAXDC2 and MSMO1 reduces total cholesterol levels and prevents cellular proliferation. HPAF-II cells, HPAF-II cells with doxycycline-inducible (DOX-inducible) single-guide (isg) RNAs targeting *FAXDC2* or *MSMO1* alone, and *FAXDC2*-KO cells with DOX-isg targeting *MSMO1* were cultured for 10 days. (**E**) Representative images of crystal violet staining from 3 independent experiments. (**F**) Crystal violet dye was solubilized, and absorbance was measured at 570 nm. (**G**) Total cholesterol levels were significantly reduced in the FAXDC2 and MSMO1 double-KO cells compared with the single knockouts. (**H** and **I**) ETC-159 treatment reduces C4-methyl sterols in HPAF-II orthotopic tumors with high FAXDC2 expression. Sterol levels in mice treated with vehicle or ETC-159 were measured by GC-MS. Data from individual tumor samples (*n* = 4–5 per group) from 2 independent experiments were combined to calculate *P* values, controlling for batch effects. (**J**–**L**) Stabilized β-catenin, which represses FAXDC2, increased C4-methyl sterol levels independent of upstream Wnt signaling inhibition. C4-methyl sterol levels in HPAF-II tumors with and without stabilized β-catenin from mice treated with ETC-159 or vehicle were analyzed by GC-MS. Each point denotes an individual tumor, *n* = 4–5 per group. (**M**) C4-methyl sterols are reduced in TCF7L2-KO HCT116 xenografts compared with control tumors. Sterol levels were measured by GC-MS. Each point depicts an individual tumor sample. *P* values were calculated by Mann-Whitney *U* test (**G** and **M**) and unpaired *t* test (**J**–**L**). *P ≤ 0.05, **P ≤ 0.01, ***P ≤ 0.001.

During cholesterol biosynthesis, squalene is oxidized and cyclized to form lanosterol, a C4-dimethyl sterol (Figure 2, A and B). Conversion of lanosterol to cholesterol proceeds down 2 parallel routes known as the Bloch and Kandutsch-Russell (KR) pathways and involves 9 distinct enzymes (Figure 2A) (18, 30, 31). One of the key steps in this process is the removal of the 2 methyl groups at the C4 position. The oxidative removal of these C4 methyl groups in the endoplasmic reticulum is catalyzed in a stepwise series of reactions (Figure 2, A and B) by methyl sterol monooxygenase 1 (MSMO1), NAD(P)-dependent steroid dehydrogenase–like (NSDHL), and keto-reductase hydroxysteroid 17β-dehydrogenase 7 (HSD17B7) (32, 33).

FAXDC2 is annotated as a paralog of MSMO1 (Alliance of Genome Resources HGNC:1334, version 5.3.0) (34) but has not been extensively studied. Consistent with the annotation, FAXDC2 has 27.5% amino acid identity and 49% similarity with MSMO1 and 24.2% identity with another C4 methyl sterol oxidase, ERG25 in *Saccharomyces cerevisiae* (Supplemental Figure 2A). All contain the 3 conserved histidine-rich motifs (HX3H, HX2HH, and HX2HH) characteristic of membrane-bound non-heme iron oxygenases (35). Orthologs of FAXDC2 are similarly conserved in plants and sponges (Supplemental Figure 2A) (36, 37). The AlphaFold-predicted structures (38) of MSMO1 and FAXDC2 are nearly superimposable with an RMSD of 1.4 Å (Figure 2C). Finally, previous studies in plants show that 2 orthologs of MSMO1 cooperate with NSDHL and other enzymes critical for the demethylation of cholesterol biosynthesis intermediates. Consistent with FAXDC2 being a paralog of MSMO1 and acting as a C4-demethylase, immunofluorescence experiments to interrogate the interactions of enzymes of the demethylase complex showed that FAXDC2, like MSMO1, can colocalize with NSDHL in the smooth ER (Figure 2D and Supplemental Figure 2B). Thus, sequence homology, predicted structure, and intracellular localization are all consistent with FAXDC2 being a C4-methyl sterol oxidase.

If FAXDC2 is a functional paralog of MSMO1 in the cholesterol biosynthesis pathway, cells lacking either MSMO1 or FAXDC2 should have reduced cholesterol synthesis and impaired cell growth in medium with delipidated cholesterol-poor serum. Moreover, the knockout of both enzymes should turn off both the KR and Bloch arms of the cholesterol biosynthesis pathway and further compromise cell growth. *FAXDC2* knockout alone in HPAF-II cells (where expression of *FAXDC2* is already repressed/low) did not reduce cell viability as assessed by colony formation (Figure 2, E and F). In contrast, inducible knockout of *MSMO1* caused an approximately 40% decrease in colony formation, suggesting that these cells were more reliant on flux through the Bloch pathway. Notably, inducible knockout of *MSMO1* in cells lacking *FAXDC2* led to an approximately 90% decrease in colony-forming ability. Under these conditions, total cellular cholesterol levels were markedly decreased by *MSMO1* knockout and further reduced in cells lacking both *MSMO1* and *FAXDC2* (Figure 2G). This is consistent with FAXDC2 being a paralog of MSMO1 in the cholesterol biosynthesis pathway.

### FAXDC2 regulates the abundance of C4-methyl sterols.

To test the function of FAXDC2 as a C4-methyl sterol oxidase involved in cholesterol biosynthesis, we postulated that there should be differences in sterol composition between the control FAXDC2-low (Wnt-high) and ETC-159–treated, FAXDC2-high (Wnt-low) HPAF-II tumors. To test this, we measured the abundance of cholesterol biosynthesis intermediates in the vehicle- and ETC-159–treated tumors from 2 independent experiments. Gas chromatography–mass spectrometry (GC-MS) analysis identified three C4-methyl sterols in FAXDC2-low HPAF-II orthotopic xenografts: 4α-methylcholest-7-enol (lophenol), 4,4-dimethylcholest-8-enol (dihydro-T-MAS), and 4,4-dimethylcholesta-8,24-dienol (T-MAS) (Supplemental Figure 3, A and B), (18). The abundance of all three C4-methyl sterols significantly decreased as FAXDC2 abundance increased in the tumors treated with the Wnt inhibitor (Figure 2, H and I). These biochemical data suggest that FAXDC2 catalyzes a rate-limiting C4-demethylation of specific cholesterol biosynthesis intermediates. High levels of FAXDC2 can drive enhanced flux through the cholesterol biosynthesis pathway and hence reduce the abundance of lophenol, dihydro-T-MAS, and T-MAS, consistent with FAXDC2 being a methyl sterol oxidase.

Our model predicts that ectopically stabilized β-catenin, by repressing FAXDC2 expression (Figure 1E), should block the ETC-159–induced changes in cholesterol biosynthesis and prevent changes in the abundance of C4-methyl sterols. Consistent with this, tumors with stabilized β-catenin had low FAXDC2 (Figure 1E) and higher basal levels of lophenol and dihydro-T-MAS than the HPAF-II tumors (Figure 2, J–L). Furthermore, the abundance of these C4-methyl sterols was unaltered by ETC-159 treatment in HPAF-II tumors with stabilized β-catenin, confirming that the drug acts on cholesterol biosynthesis through its effects on the Wnt/β-catenin pathway.

In HCT116 colorectal cancer xenografts that have hyperactive β-catenin and low *FAXDC2*, we also observed accumulation of C4-methyl sterols lophenol and dihydro-T-MAS. In these xenografts, genetic inhibition of β-catenin activity by TCF7L2 knockout also significantly reduced the abundance of the sterols, again inversely correlated with *FAXDC2* expression (Figure 1F and Figure 2M).

The two C4-methyl sterols that are most robustly decreased upon Wnt inhibition, lophenol and dihydro-T-MAS, being saturated at C24 (Figure 2B), are specific to the KR arm of the cholesterol biosynthesis pathway. The key difference between the Bloch and the KR pathway is the step at which the C24 double bond is reduced by 24-dehydrocholesterol reductase (DHCR24). The Bloch pathway utilizes DHCR24 in the last step of cholesterol biosynthesis, while the KR pathway uses DHCR24 to convert lanosterol to 24,25-dihydrolanosterol much earlier in the biosynthesis of cholesterol (Figure 2A). This flow is largely unidirectional such that the Bloch pathway intermediates may cross over into the KR pathway at multiple steps but not necessarily vice versa (31). The correlation of changes in 24,25-dihydro-C4-methyl sterols in HPAF-II xenografts with FAXDC2 levels suggests that FAXDC2 is a methyl sterol oxidase predominantly in the KR pathway and that it catalyzes the steps between dihydro-T-MAS and zymostenol, parallel to the activity of MSMO1 in the Bloch pathway (Figure 2, A and B). Low FAXDC2 activity in Wnt-high tumors causes an accumulation of upstream C4-methyl sterols of the KR pathway. In contrast, an increase in FAXDC2 activity in Wnt-inhibited tumors enhances flux through this branch of the pathway, leading to the depletion of C4-methyl sterols (lophenol and dihydro-T-MAS) upstream of the steps it catalyzes (Figure 2, H and I).

Taken together, these data show that FAXDC2 is a C4-methyl sterol oxidase that is repressed by high Wnt/β-catenin signaling. FAXDC2 acts preferentially in the KR branch of the cholesterol biosynthesis pathway, and its upregulation changes flux through the pathway, thereby altering the abundance of specific C4-methyl sterols.

### FAXDC2 is repressed in Wnt-high human cancers and leads to an accumulation of C4-methyl sterols.

**Figure 3:**
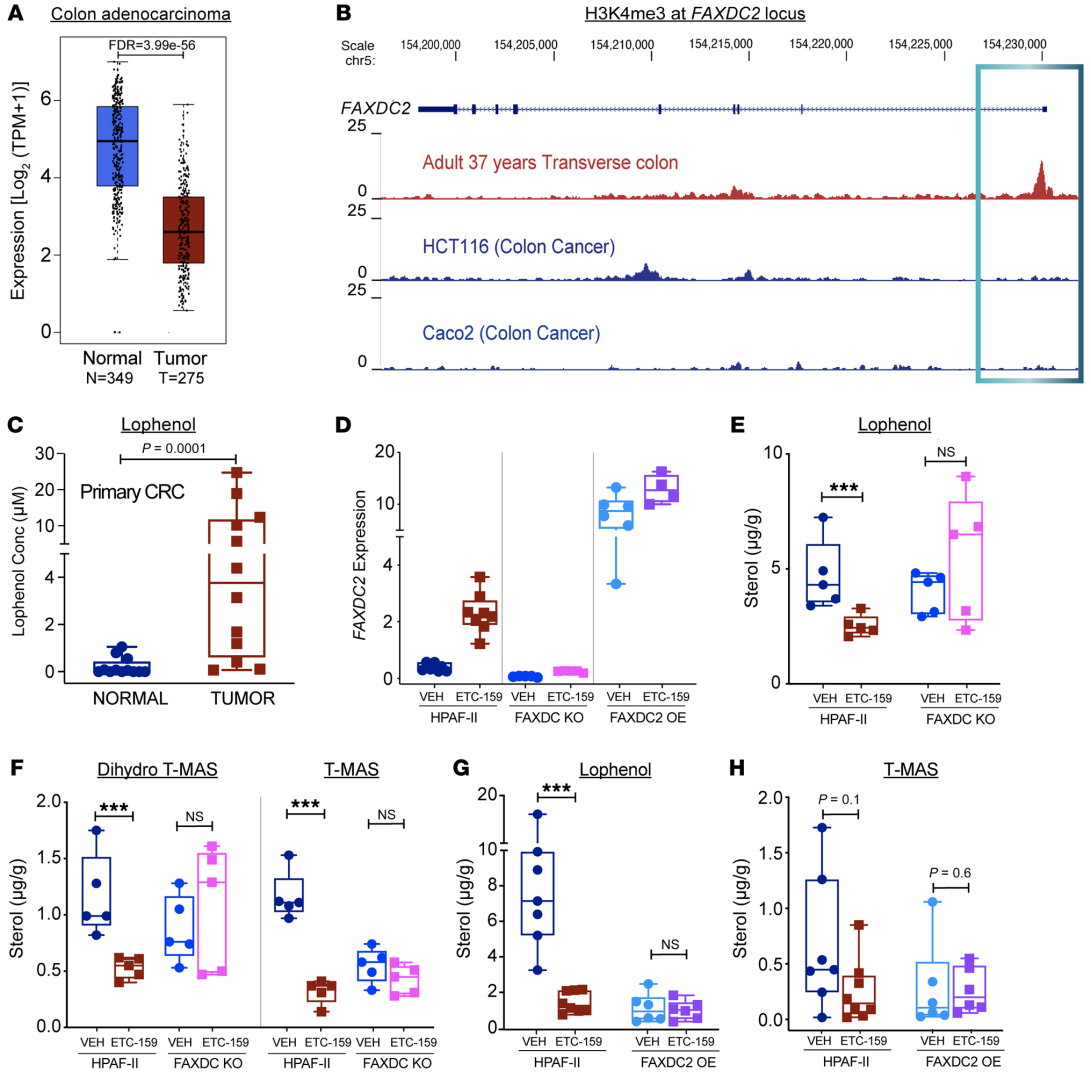
*FAXDC2* expression correlates with and drives changes in C4-methyl sterols. (**A**–**C**) Primary Wnt-high colorectal cancers have repressed FAXDC2 expression and high levels of lophenol. (**A**) FAXDC2 is repressed in primary colorectal cancers compared with corresponding normal tissues. Expression of *FAXDC2* was compared in normal tissues versus tumor tissues using GEPIA 2.0 (TCGA and GTEx data sets). (**B**) H3K4me3, a marker of active transcription, marks the *FAXDC2* genomic locus in the normal colon but is absent in cancer cell lines. Data from Cistrome Data Browser. (**C**) Lophenol is markedly elevated in primary colorectal cancers. Sterol abundance was measured in primary colorectal cancers and adjacent normal tissue by GC-MS. Each point represents lophenol levels in an individual tumor, *n* = 12 tumors per group. (**D**–**H**) Manipulation of FAXDC2 expression regulates C4-methyl sterol abundance. (**D**) Relative expression of *FAXDC2* in HPAF-II, FAXDC2-KO, and FAXDC2-OE xenografts as measured by qRT-PCR. (**E** and **F**) Knockout of FAXDC2 abrogates the ETC-159 treatment–induced change in the abundance of C4-methyl sterols. Sterol abundance was measured in HPAF-II and FAXDC2-KO xenografts from control and ETC-159–treated mice. Each point represents the level of indicated methyl sterols measured using GC-MS in an individual tumor sample, *n* = 4–5 per group. (**G** and **H**) FAXDC2 overexpression reduces lophenol and T-MAS to levels comparable to those in the Wnt-inhibited tumors. Sterol abundance was measured in HPAF-II and FAXDC2-OE xenografts from control and ETC-159–treated mice. Each point represents the abundance of methyl sterols measured using GC-MS in an individual tumor sample, *n* = 4–5 per group. *P* values were calculated by Mann-Whitney *U* test for all graphs in this figure. ***P ≤ 0.001.

Pathologic Wnt activation occurs in over 80% of human colorectal cancers. To examine whether *FAXDC2* was repressed in Wnt-high cancers, we analyzed the data from Gene Expression Profiling Interactive Analysis (GEPIA 2.0) (39). Consistent with our xenograft data, *FAXDC2* expression was significantly repressed in human colorectal cancers compared with normal colorectal tissue (Figure 3A). Additionally, promoter analysis using data sets from the Encyclopedia of DNA Elements (ENCODE) (40) showed that the H3K4me3 activation mark was present on the promoter of *FAXDC2* in normal colon tissue but was completely absent in 2 colon cancer cell lines (Figure 3B).

If *FAXDC2* is repressed in most human colorectal cancers, then methyl sterol intermediates should accumulate in those cancers. To test this, we measured both lophenol abundance and *FAXDC2* expression in locally available matched normal and tumor tissues from Wnt-high colorectal cancer patients. Consistent with the GEPIA data, in 50 paired normal and tumor samples from colorectal cancer patients, *FAXDC2* expression was significantly lower in the tumors compared with the normal tissues (Supplemental Figure 4A). Most strikingly, these colorectal cancers had markedly elevated levels of lophenol compared with its low abundance in the matched normal tissues with high *FAXDC2* levels (Figure 3C). These data are similar to our findings in Wnt-high pancreatic cancer xenografts and support the model that elevated Wnt/β-catenin signaling represses *FAXDC2* expression, decreasing flux through the KR branch of the cholesterol biosynthesis pathway, leading to the accumulation of upstream intermediates, including lophenol and other C4-methyl sterols.

### FAXDC2 mediates Wnt-regulated changes in C4-methyl sterols.

Our data show a strong inverse correlation between FAXDC2 levels and C4-methyl sterol abundance. To directly test whether FAXDC2 is required for the changes in sterol abundance, we used CRISPR/Cas9 to create FAXDC2-knockout clones using sgRNAs (Supplemental Figure 4, B and C). HPAF-II WT or *FAXDC2*-KO cells were injected into mice to generate xenograft tumors. The absence of FAXDC2 transcript and protein in the KO tumors was confirmed by quantitative reverse transcription PCR and immunoblots (Figure 3D and Supplemental Figure 4D). *FAXDC2* expression and C4-methyl sterol abundance were measured before and after 7 days of Wnt inhibition. Knockout of *FAXDC2* abrogated the increase in *FAXDC2* expression induced by Wnt inhibition (Figure 3D). Notably, compared with the control tumors, *FAXDC2* knockout blocked the Wnt inhibition–mediated decrease in the C4-methyl sterols of the KR pathway, lophenol, and dihydro-T-MAS (Figure 3, E and F). This shows directly that the absence of FAXDC2 causes an accumulation of C4-methyl sterols; hence it is required for their demethylation.

Next, we rescued the expression of *FAXDC2* in the KO cells by stably expressing it under the control of a CMV promoter (hereafter referred to as *FAXDC2*-OE cells). The xenografts from these cells had a 10-fold higher than normal expression of *FAXDC2* (Figure 3D). As predicted, the *FAXDC2*-OE xenografts from the vehicle-treated mice had markedly reduced levels of lophenol and T-MAS, comparable to what was seen following Wnt inhibition in HPAF-II tumors when FAXDC2 levels increased (Figure 3, G and H). Notably, *FAXDC2*-OE tumors showed no further decrease in sterol abundance following Wnt inhibition, directly demonstrating that FAXDC2 regulates flux through the cholesterol biosynthesis pathway and mediates Wnt-regulated changes in C4-methyl sterol abundance. Taken together, these studies establish that FAXDC2 is a Wnt-repressed enzyme in the KR branch of the cholesterol biosynthesis pathway that regulates the abundance of 24-saturated C4-methyl sterols.

### FAXDC2 activity influences Wnt-regulated gene expression.

**Figure 4:**
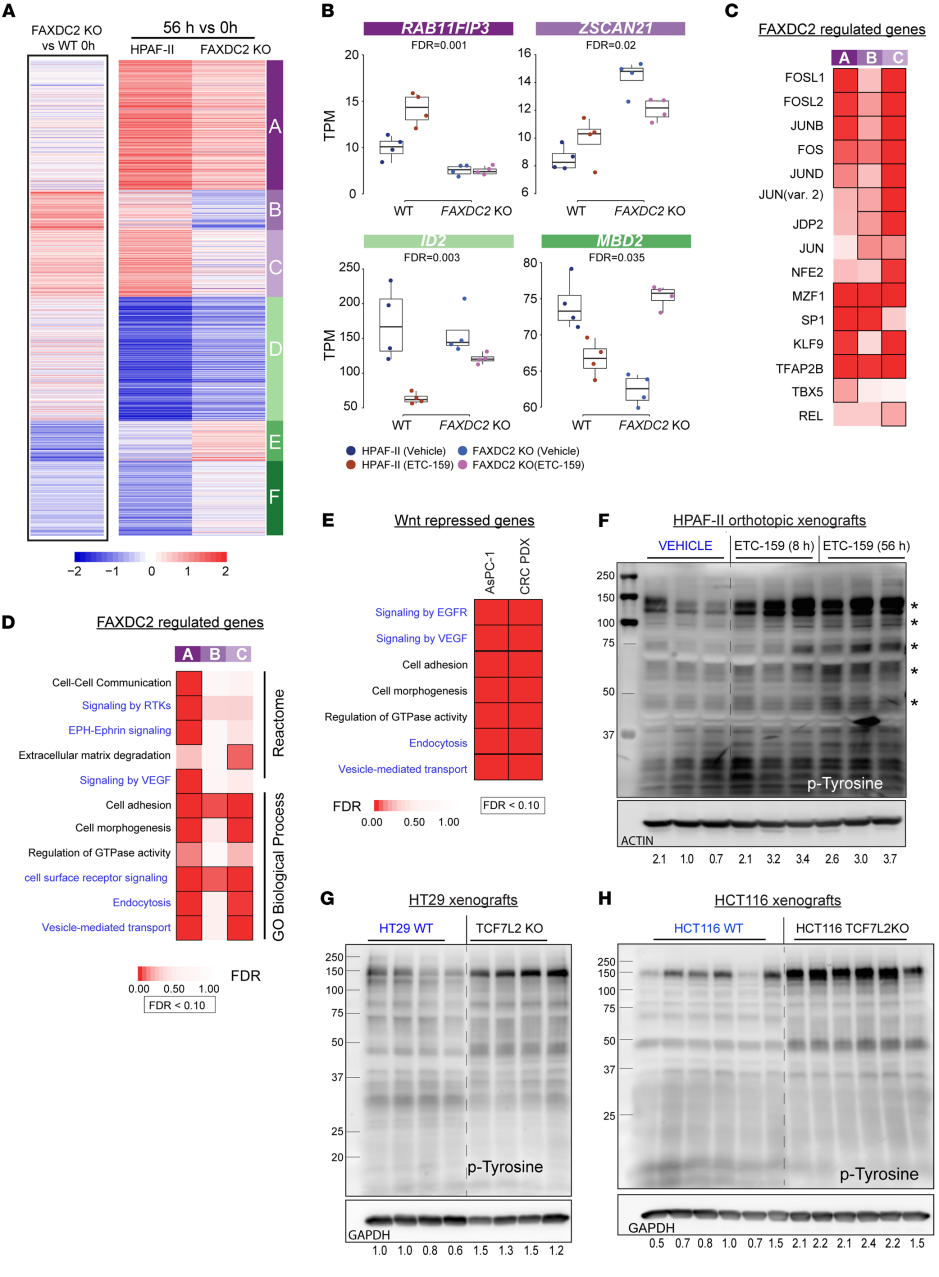
FAXDC2 is a downstream effector of Wnt signaling, and *FAXDC2*-regulated genes are enriched for RTK signaling. (**A**) Expression of a subset of Wnt-regulated genes depends on FAXDC2 (FDR < 10%). Heatmap of fold changes of 3,560 *FAXDC2*-dependent Wnt-regulated genes (interaction test, FDR < 10%). Left: Gene expression changes (log_2_ fold changes) between WT and FAXDC2-KO tumors at 0 hours. Right: Gene expression changes following treatment with ETC-159 in both parental HPAF-II tumors (56 vs. 0 hours) and FAXDC2-KO tumors (56 vs. 0 hours). This set of differentially responding genes was partitioned into 6 distinct clusters based on similarities in their response to ETC-159, including 3 clusters of Wnt-repressed genes (A, B, and C) and 3 three clusters of Wnt-activated genes (D, E, and F). (**B**) Representative genes from 4 clusters in part **A** show that FAXDC2 knockout blunts or reverses the response to Wnt inhibition (hypergeometric test, FDR < 10%). TPM, transcripts per million. (**C**) FAXDC2-regulated Wnt-repressed genes in clusters A–C show an enrichment of AP1 family TFBS motifs in their promoters (hypergeometric test, FDR < 10%). (**D**) RTK signaling pathways are enriched in FAXDC2-regulated Wnt-repressed genes. Data from representative GO Biological Process and Reactome enrichment of Wnt-repressed genes from clusters A–C that were identified in part **A** (hypergeometric test, FDR < 10%). (**E**) GO Biological Processes and Reactome enrichment analysis of Wnt-repressed genes in ETC-159–treated AsPC-1 and colorectal cancer PDX tumors shows an upregulation of RTK signaling pathways (hypergeometric test, FDR < 10%). (**F**) Pharmacologic Wnt inhibition with ETC-159 in HPAF-II xenografts increases protein tyrosine phosphorylation. HPAF-II orthotopic tumor protein lysates from vehicle- or ETC-159–treated mice were separated on a 10% SDS gel. Blots were probed with phosphotyrosine antibodies. Each lane represents tumor lysate from an individual mouse. Asterisks indicate prominent phospho-tyrosine bands. (**G** and **H**) Genetic inhibition of Wnt/β-catenin signaling in TCF7L2-KO HT29 and HCT116 xenografts increases protein tyrosine phosphorylation. Protein lysates from WT and *TCF7L2-*KO HT29 and HCT116 tumors were analyzed for abundance of phosphotyrosine proteins as in **F**. Each lane contains tumor lysate from an individual mouse.

While acting as precursors for cholesterol, C4-methyl sterols also act as signaling molecules in major biological processes, including auxin transport in plants, dauer formation in *C*. *elegans*, oogenesis, and oligodendrocyte differentiation in mammals (21, 22, 41). We speculated that FAXDC2-regulated changes in C4-methyl sterols might also regulate downstream signaling pathways. To test this, we assessed the transcriptional landscape of HPAF-II xenografts with or without *FAXDC2* knockout (Figure 4A, left). Knockout of *FAXDC2* altered the expression of 3,570 genes compared with the parental HPAF-II tumors (fold change > 1.5, FDR < 10%), consistent with a significant role for C4-methyl sterols in cellular signaling pathways. These FAXDC2-dependent genes were grouped into 6 clusters (Figure 4A and Supplemental Figure 5, A and B).

As noted, Wnt inhibition regulates the expression of 11,673 genes in HPAF-II orthotopic xenografts (Supplemental Figure 1A). The response of 2,159 of the Wnt-regulated genes in HPAF-II xenografts depended on FAXDC2. FAXDC2 regulated 1,146 Wnt-repressed genes, i.e., genes that were upregulated by Wnt inhibition in the HPAF-II WT tumors but were unaltered in *FAXDC2*-KO tumors (Figure 4A, clusters A–C, and Supplemental Table 1) (FDR < 10%, interaction test), and 1,013 Wnt-activated genes (Figure 4A, clusters D–F). Examples of such FAXDC2-regulated genes, *RAB11FIP3*, *IGFBP3*, *ID2*, and *MBD2*, are shown in Figure 4B and Supplemental Figure 5C. Thus, FAXDC2 is essential for regulating a significant proportion of the genes whose expression changes upon Wnt inhibition.

To better understand the signaling pathways regulated by FAXDC2, we performed transcription factor binding site analysis of the FAXDC2-regulated genes and found enrichment for ETS and AP1 binding sites (Figure 4C and Supplemental Table 2). ETS and AP1 transcription factors act downstream of MAPK signaling to regulate gene transcription, suggesting a role for FAXDC2 in regulating MAPK signaling. This is consistent with our previous reports that Wnt inhibition activates MAPK signaling (16, 17). Gene Ontology (GO) and Reactome pathway analysis of these FAXDC2-dependent genes also showed significant enrichment for genes regulating vesicle-mediated transport, signaling by receptor tyrosine kinases (RTKs), and cell-cell communication processes (Figure 4D). A similar enrichment was also present in Wnt-regulated genes in all 3 Wnt ligand–addicted models, HPAF-II and AsPC-1 orthotopic xenografts and colorectal cancer PDX (Figure 1A and Figure 4E). This is consistent with FAXDC2 being a major downstream effector of Wnt signaling and suggests that Wnt signaling, via FAXDC2, could regulate RTKs and downstream MAPK signaling.

### Wnt inhibition leads to an increase in RTK activation in Wnt-high tumors.

To examine whether Wnt inhibition activated protein tyrosine kinases, we analyzed changes in tyrosine phosphorylation in the vehicle- and ETC-159–treated HPAF-II tumor xenografts. Remarkably, Wnt inhibition led to a robust increase in phosphotyrosine staining of multiple proteins in tumor lysates (Figure 4F). The increase in tyrosine phosphorylation of multiple proteins in response to Wnt inhibition was confirmed by a phosphotyrosine array (Supplemental Figure 5D). This shows that tyrosine kinase signaling is activated upon Wnt inhibition in these tumors. Similarly, genetic inhibition of Wnt/β-catenin signaling (by knockout of *TCF7L2*) caused enhanced tyrosine phosphorylation in 2 additional models, APC-mutant HT29 and β-catenin mutant HCT116 colorectal cancer xenografts (Figure 4, G and H). Thus, inhibiting Wnt/β-catenin signaling either pharmacologically or genetically leads to the activation of cellular tyrosine phosphorylation.

### Wnt inhibition increases the recycling of RTKs.

**Figure 5:**
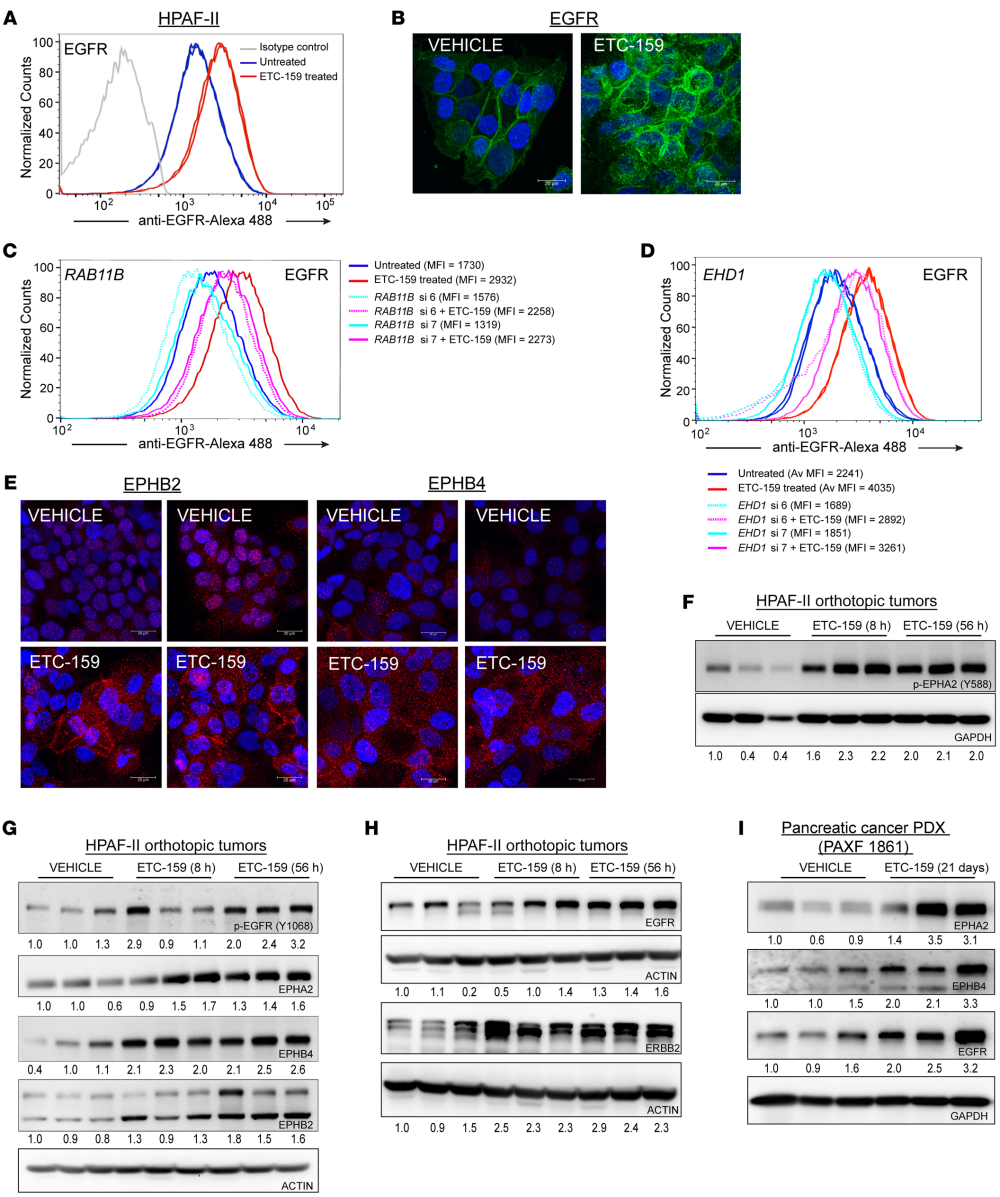
Increased RTK recycling in Wnt-addicted cancers upon Wnt inhibition. (**A** and **B**) ETC-159 treatment of HPAF-II cells increases abundance of EGFR on the cell surface. HPAF-II cells were treated with 100 nM ETC-159 for 72 hours. (**A**) Cells were stained with Alexa Fluor 488–conjugated anti-EGFR antibody and analyzed by flow cytometry. Each histogram represents ~50,000 cells. Data are representative of 3 independent experiments (*P* = 0.009). (**B**) Endogenous EGFR levels on non-permeabilized cells were assessed by indirect immunofluorescence microscopy. Scale bars: 20 μm. (**C** and **D**) Partial knockdown of RAB11B or EHD1 blunts the ETC-159–induced EGFR increase on the surface of HPAF-II cells. HPAF-II cells were transfected with 2 independent siRNAs (si6 and si7) against *RAB11B* (**C**) or *EHD1* (**D**) for 24 hours, followed by treatment with ETC-159 for 72 hours. EGFR levels on the cell surface were assessed by flow cytometry. Each histogram represents ~50,000 cells from 1 replicate. Data are representative of 3 independent experiments. Average median fluorescence intensity (MFI) of the technical replicates is shown. Av MFI, average MFI of the technical replicates from the same experiment. (**E**) EPHB2 and EPHB4 levels are increased by Wnt inhibition. HPAF-II cells were treated with 100 nM ETC-159 for 72 hours, and the levels of endogenous EPHB2 and EPHB4 on non-permeabilized cells were assessed by indirect immunofluorescence microscopy. Scale bars: 20 μm. (**F**–**I**) Wnt inhibition increases activation of EPHA2 and EGFR receptor tyrosine kinases and protein abundance of multiple receptor tyrosine kinases in HPAF-II xenografts and pancreatic PDX models. Protein lysates from HPAF-II orthotopic xenografts or pancreatic PDX from vehicle- or ETC-159–treated mice were analyzed for expression of p-EPHA2 and p-EGFR and abundance of indicated RTKs by Western blots. Each lane represents tumor lysate from an individual mouse. (**H**) The protein lysates were prepared as a master mix and loaded on independent gels. Only 1 blot was probed for the load control, which is shared with Figure 4F.

RTK protein abundance and activity are regulated by a balance of endocytosis, recycling, and degradation (42, 43). GO and Reactome analysis indicates that Wnt signaling regulates receptor recycling. Reduced endocytosis and lysosomal degradation lead to increased cell surface abundance of RTKs, where they are available for interaction with their ligands and activation of downstream signaling. We therefore tested whether Wnt inhibition increased the cell surface abundance of EGFR, a prototype for RTK endocytic recycling. Indeed, the abundance of EGFR on the surface of cultured Wnt ligand–dependent pancreatic cancer cell lines HPAF-II and AsPC-1 increased significantly following Wnt inhibition (Figure 5A and Supplemental Figure 6A). Likewise, a clear relocalization of EGFR to the cell membrane following ETC-159 treatment was seen by indirect immunofluorescence staining of non-permeabilized HPAF-II cells (Figure 5B). These data indicate that enhanced EGFR recycling to the plasma membrane upon Wnt inhibition is a general phenomenon in Wnt ligand–dependent cancers.

To further assess the role of endocytic recycling in regulating Wnt inhibition–mediated changes in cell surface EGFR abundance, we knocked down multiple genes associated with recycling endosomes (44, 45). While knockdown of *RAB4*, *RAB25*, and *RAB11A* had no effect, knockdown of *RAB11B* using 2 independent siRNAs substantially reduced the ETC-159–mediated increase in EGFR levels on the surface of HPAF-II cells (Figure 5C and Supplemental Figure 6, B–H) (46, 47). Like *RAB11B* knockdown, the knockdown of *EHD1* using 2 independent siRNAs also mitigated the ETC-159–mediated increase in the cell surface abundance of EGFR in HPAF-II cells (Figure 5D and Supplemental Figure 6I) (48). Taken together, these data suggest that enhanced late endocytic recycling leads to an increase in cell surface abundance of EGFR following Wnt inhibition.

RAB7 is a key Rab required for the transport from late endosomes to lysosomes. As can be seen in Supplemental Figure 6, J and K, there was no effect of RAB7 on the Wnt inhibition–mediated increase in EGFR recycling, suggesting that this change is not driven by diminished trafficking to lysosomes.

Enhanced endosomal recycling to the plasma membrane can increase the total and cell surface abundance of multiple receptor tyrosine kinases (43). To test whether Wnts regulate the recycling of multiple RTKs, we used flow cytometry and indirect immunofluorescence staining of non-permeabilized HPAF-II cells. Indeed, we observed an increase in EPHA2, EPHB2, and EPHB4 on the surface of cultured HPAF-II cells (Figure 5E and Supplemental Figure 6, L and M) after Wnt inhibition. The increase in the ephrin receptors at the cell surface is biologically relevant given their known role in providing positional cues for differentiation (49).

Next, we examined HPAF-II tumor lysates from mice treated with ETC-159 and observed an increase in the phosphorylation of EGFR at Y1068 and EPHA2 at Y588. This confirms that Wnt inhibition leads to the activation of EGFR and EPHA2 in vivo (Figure 5, F and G). We were unable to measure changes in the phosphorylation of other Eph receptors because of the unavailability of reliable antibodies.

As a consequence of increased recycling, the total protein abundance of multiple RTKs was also increased following Wnt inhibition in HPAF-II orthotopic xenografts (Figure 5, G and H). We observed an increase in the protein abundance of EGFR, ERBB2, EPHA2, EPHB4, and EPHB2, while many of these genes had no change in transcript abundance (Figure 5, G and H, and Supplemental Figure 6, N and O). This increase in the protein abundance of RTKs following Wnt inhibition was observed in multiple models, as there was also an increase in the protein abundance of ERBB2, EGFR, and multiple EPH receptors in PAXF1861 pancreatic patient-derived subcutaneous xenografts with an RNF43 G371fs mutation (Figure 5I). Notably, unlike what is observed in the xenografts, despite the increase in cell surface RTKs, there was minimal activation of phosphotyrosine following Wnt inhibition in cultured cells. Taken together, our data indicate that Wnt signaling can regulate the recycling of multiple RTKs from late endosomes to the plasma membrane. The RTKs can then be activated by ligands present only in the local tumor environment.

### FAXDC2 regulates Wnt inhibition–mediated changes in RTK abundance.

The cholesterol biosynthesis enzyme MSMO1, a C4-methyl sterol oxidase in the Bloch pathway, has previously been shown to regulate EGFR signaling in breast cancer cells (23, 50). To test whether the RTK activation was dependent on changes in sterol metabolism in Wnt-high cells, we examined the effect of the CYP51A1 inhibitor ketoconazole. Ketoconazole leads to the depletion of cholesterol biosynthesis intermediates downstream of lanosterol. Similarly to what was observed with ETC-159 treatment, ketoconazole increased cell surface levels of EGFR in HPAF-II cells (Supplemental Figure 7A), suggesting the importance of sterols in regulating RTK signaling in these cells. FAXDC2 changes the abundance of C4-methyl sterols, and FAXDC2-dependent genes were significantly enriched for endocytosis and vesicle-mediated transport processes (Figure 4D). We therefore tested with 3 independent approaches whether FAXDC2 regulates RTK signaling in Wnt-addicted pancreatic cancer models.

**Figure 6:**
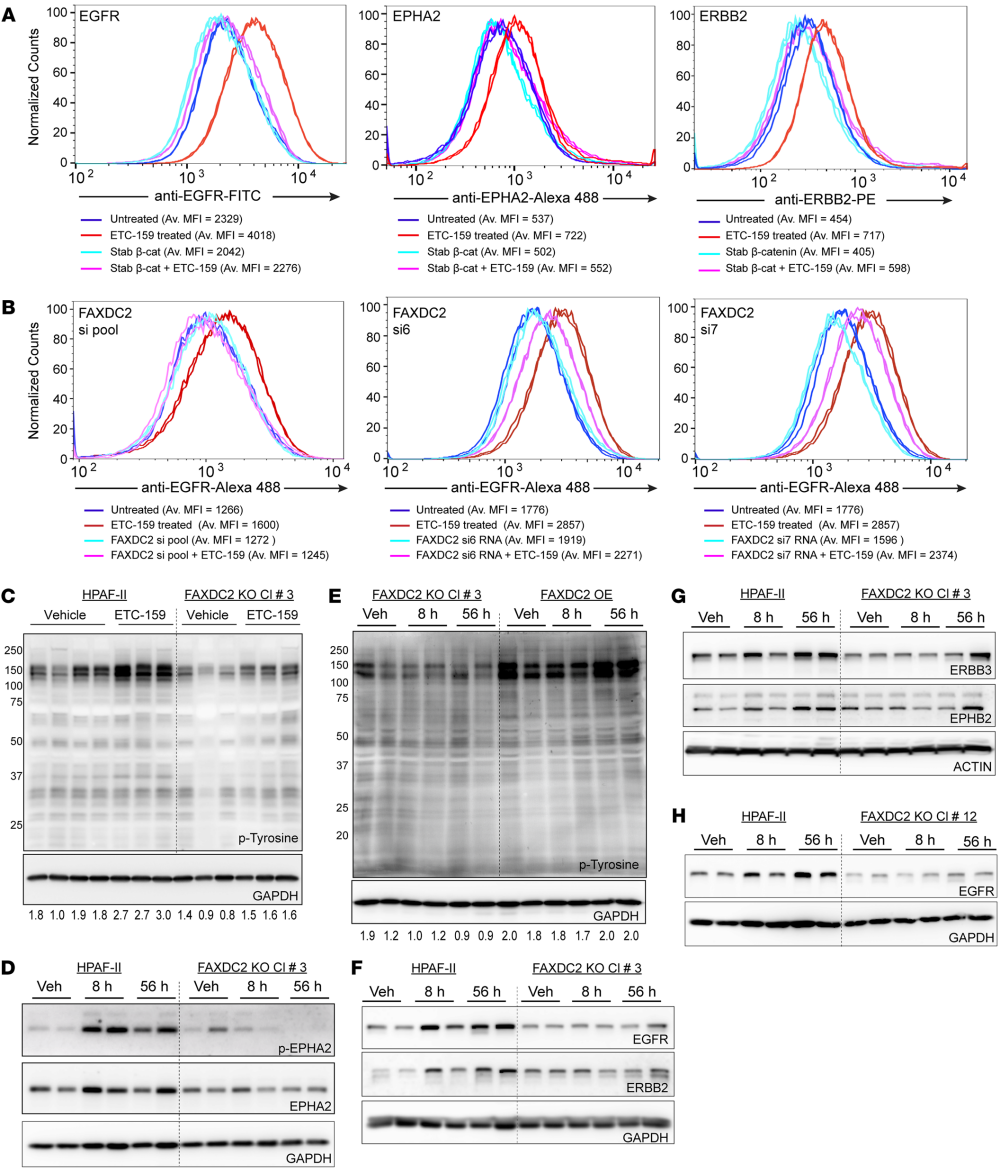
*FAXDC2* is downstream of Wnt/β-catenin in the regulation of RTK signaling. (**A**) Stabilized β-catenin prevents multiple RTKs from moving to the cell surface upon treatment with ETC-159. Cell surface abundance of EGFR, ERBB2, and EPHA2 was assessed as before in HPAF-II cells with or without stabilized β-catenin. Cells were treated with DMSO or 100 nM ETC-159 for 72 hours before staining with fluorescent tagged antibodies. Each histogram represents ~50,000 cells. Data are representative of 3 independent experiments, and 2 replicates are shown. (**B**) Knockdown of FAXDC2 prevents Wnt inhibition–mediated increase in EGFR cell surface abundance. HPAF-II cells were transfected with a pool of 4 siRNAs or 2 independent siRNAs (si6 and si7) against *FAXDC2* for 24 hours, followed by treatment with ETC-159 for 72 hours. The cells were then stained with Alexa Fluor 488–conjugated anti-EGFR antibody and analyzed by flow cytometry. Data are representative of 3 independent experiments, and 2 replicates are shown. (**C** and **D**) Knockout of FAXDC2 in HPAF-II tumors prevents Wnt inhibition–mediated increase in tyrosine phosphorylation and p-EPHA2 levels. Protein lysates from HPAF-II or FAXDC2-KO tumor xenografts from vehicle- or ETC-159–treated mice were separated on 10% SDS gels and transferred to a PVDF membrane. Membranes were probed with anti-phosphotyrosine or –p-EphA2 antibodies. Each lane represents lysate from an individual tumor. (**E**) Overexpression of FAXDC2 in FAXDC2-KO tumors rescues the Wnt inhibition–mediated increase in tyrosine phosphorylation. Protein lysates from FAXDC2-KO or FAXDC2-OE tumor xenografts from vehicle- or ETC-159–treated mice were probed with phosphotyrosine antibodies. Each lane represents lysate from an individual tumor. (**F**–**H**) FAXDC2 knockout prevents the Wnt inhibition–mediated increase in EGFR and Eph family receptor tyrosine kinase abundance. Protein lysates from HPAF-II or FAXDC2-KO tumors from mice treated with vehicle or ETC-159 were analyzed as in **C**. Data are shown from 2 independent FAXDC2-KO clones, clone 3 (**F** and **G**) and clone 12 (**H**). Each lane represents lysate from an individual tumor.

First, we used cells with stabilized β-catenin that have low levels of FAXDC2 and high levels of lophenol and dihydro-T-MAS even upon ETC-159 treatment (Figure 1E and Figure 2, K and L). If FAXDC2 regulates RTK activation, we predicted that stabilized β-catenin would prevent the ETC-159–mediated increase in cell surface levels of RTKs. Indeed, stabilized β-catenin abrogated the increase in cell surface levels of multiple RTKs, including EGFR, EPHA2, and ERBB2, in HPAF-II cells and xenografts treated with ETC-159 (Figure 6A and Supplemental Figure 7B).

Second, we tested the direct effect of *FAXDC2* knockdown on EGFR levels in vitro. HPAF-II cells were transfected with a pool of siRNAs or 2 independent guide RNAs targeting *FAXDC2* in the presence or absence of ETC-159. As expected, Wnt inhibition increased cell surface EGFR levels, but the knockdown of *FAXDC2* prevented this increase in EGFR (Figure 6B and Supplemental Figure 7, C and D). Notably, we observed that siRNA knockdown of *MSMO1* did not affect ETC-159–mediated changes in EGFR cell surface levels in HPAF-II cells (Supplemental Figure 7, E and F), suggesting that the observed effect is not a consequence of changes in overall cholesterol levels. This implies that FAXDC2 regulates cell surface receptor levels following Wnt inhibition in Wnt ligand–addicted models through its effect on KR-specific methyl sterols.

Third, we tested whether FAXDC2 is required for the Wnt inhibition–mediated increase in RTK signaling in vivo. As before, there was a marked increase in tyrosine phosphorylation upon Wnt inhibition in HPAF-II tumors (Figure 6C). Notably, in the absence of FAXDC2, the basal level of tyrosine phosphorylation was decreased, and the Wnt inhibition–mediated increase in tyrosine phosphorylation was also prevented (Figure 6C). We confirmed this result by examining EPHA2 tyrosine phosphorylation. EPHA2 was phosphorylated upon Wnt inhibition in parental tumors, but this increase in EPHA2 tyrosine phosphorylation was abrogated in the FAXDC2-KO tumors (Figure 6D). This effect of FAXDC2 knockout on RTK activation was so unexpected that to rule out off-target effects, we compared the phosphotyrosine levels in the FAXDC2-OE xenografts (FAXDC2-KO cells with FAXDC2 ectopically expressed under the control of CMV promoter). Consistent with a key role for FAXDC2 in regulating tyrosine phosphorylation, the FAXDC2-OE xenografts had enhanced tyrosine phosphorylation at baseline, and there was no further increase following Wnt inhibition (Figure 6E). Thus, changing FAXDC2 activity changes RTK activity.

Consistent with FAXDC2 being upstream of RTK recycling and signaling, FAXDC2 knockout blocked the effect of Wnt inhibition on the protein abundance of multiple RTKs in the tumors, including EGFR, ERBB2, ERBB3, EPHA2, and EPHB2 (Figure 6, D, F, and G). As in tumors from clone 3, the increase in EGFR abundance after Wnt inhibition was also impaired in tumors from an independent FAXDC2-KO clone, clone 12 (Figure 6H).

These data are consistent with the model that Wnt/β-catenin signaling inhibits the expression of FAXDC2, and FAXDC2 controls the abundance of specific saturated C4-mono and -dimethyl sterols to drive multiple RTKs to the plasma membrane, where they are available for ligand interaction and activation.

### FAXDC2 is necessary and sufficient for Wnt inhibition–mediated MAPK activation.

**Figure 7:**
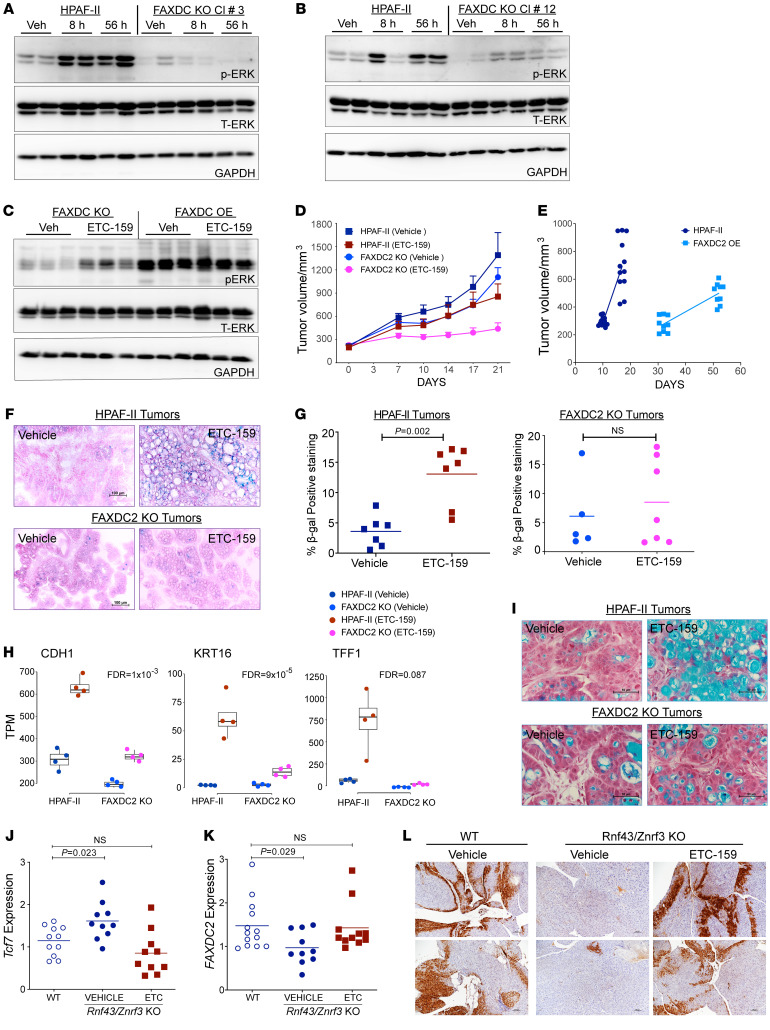
*FAXDC2* knockout prevents Wnt inhibition–induced MAPK activation, cellular differentiation, and senescence. (**A** and **B**) FAXDC2 is required for MAPK activation. Xenografts from 2 independent *FAXDC2*-KO clones had reduced ERK phosphorylation with no further increase upon ETC-159 treatment. Protein lysates were prepared as a master mix and loaded on independent gels. Load control of **A** is shared with Figure 6D and of **B** with Figure 6H. (**C**) FAXDC2 overexpression mediates sustained ERK activation. Western blot analysis of protein lysates from indicated xenografts. Each lane represents tumor lysate from an individual mouse. (**D**) Knockout of FAXDC2 reduced the growth of HPAF-II subcutaneous xenografts, with further reduction upon Wnt inhibition. *n* = 5–6 mice per group. (**E**) Overexpression of FAXDC2 delayed implantation and reduced growth of HPAF-II xenografts. *n* = 5–6 mice per group. (**F** and **G**) Wnt inhibition caused an increase in senescence-associated β-galactosidase in HPAF-II tumors that was diminished in the FAXDC2-KO tumors. (**F**) Representative images are shown. Scale bars: 100 μm. (**G**) Percentage of positively stained area (blue) was quantitated. Each dot represents quantitation of a tumor section from an individual mouse. *P* values were calculated by Mann-Whitney *U* test. (**H** and **I**) FAXDC2 knockout blunts the differentiation response to Wnt inhibition. (**H**) Expression of selected differentiation markers in tumors assessed by RNA-Seq. Each data point represents an individual tumor (hypergeometric test, FDR <10%). (**I**) FAXDC2 knockout blunts the Wnt inhibition–mediated increase in mucin expression as assessed by Alcian blue staining. Scale bars: 50 μm. (**J**–**L**) The Wnt/MAPK axis is present in non-malignant mouse pancreas. (**J** and **K**) Genetic activation and subsequent pharmacologic inhibition of Wnt signaling in mouse pancreas led to reciprocal regulation of *Faxdc2* expression. Pancreas from control or ETC-159–treated WT and *Ptf1*α*^Cre^ Rnf43^–/–^ Znrf3^–/–^* mice was analyzed for (**J**) *Tcf7* and (**K**) *Faxdc2* (Gm12248) expression by qRT-PCR. Data are from 2 independent biological experiments; each dot represents an individual mouse. *P* values were calculated by the Mann-Whitney *U* test. (**L**) Wnt activation reduced p-ERK levels in the mouse pancreas as assessed by IHC. Representative image of anti–p-ERK–stained pancreas. Scale bars: 100 μm.

RTK phosphorylation leads to the activation of downstream signaling cascades, including RAS/RAF/MEK/ERK (MAPK) signaling. Since knockout of FAXDC2 prevented the Wnt inhibition–mediated activation of multiple cellular tyrosine kinases, we tested how it impacted Wnt-regulated MAPK activation. We compared the phospho-ERK (p-ERK) levels in control and FAXDC2-KO tumors. As we previously reported, there was a significant increase in p-ERK1/2 levels upon Wnt inhibition in control tumors (Figure 7, A and B) (16). FAXDC2-KO tumors from both clone 3 and clone 12 had lower basal p-ERK than parental HPAF-II tumors (compare vehicle lanes in both groups, Figure 7, A and B). Notably, the increase in ERK phosphorylation following Wnt inhibition was nearly abrogated in the FAXDC2-KO tumors compared with the WT HPAF-II tumors (Figure 7, A and B). This was observed in 2 independent FAXDC2 knockouts, so it is neither an sgRNA nor a clonal effect. The role of FAXDC2 in MAPK activation is consistent with its effect on RTK signaling, as FAXDC2 knockout prevented the activation of RTKs. Thus, FAXDC2 expression is required for Wnt inhibition to activate both RTKs and MAPK.

To test whether ERK activation was a direct consequence of FAXDC2 expression independent of Wnt signaling, we compared the p-ERK levels in FAXDC2-OE xenografts, where both lophenol and T-MAS levels are markedly suppressed (Figure 3, G and H). Overexpression of FAXDC2 led to high basal p-ERK levels (Figure 7C), and there was no further increase upon Wnt inhibition, consistent with the effect on tyrosine phosphorylation (Figure 6E).

Taken together with the enrichment of ETS and AP1 binding sites in the genes regulated by FAXDC2, these results show that the regulation of FAXDC2 expression by the Wnt/β-catenin axis is required for maintaining endocytic recycling and activation of RTKs and downstream MAPK signaling.

### The role of FAXDC2 in Wnt inhibition–mediated senescence and differentiation.

RTK/RAS/MAPK signaling can drive proliferation, but this pathway is also essential for differentiation in multiple settings. RAS signaling in cancer is modulated for optimal growth and has a “sweet spot”; loss of activity prevents proliferation but excessive activity can cause senescence (51–53). Consistent with decreased basal p-ERK signaling, FAXDC2-KO cells grew approximately 30% slower than the parental cells both in culture and as tumors in the flanks of immunocompromised mice (Figure 7D). Conversely, FAXDC2-OE cells with markedly increased phosphotyrosine and p-ERK also grew very slowly in mice. The FAXDC2-OE cell pool took 5–6 weeks to establish tumors in the mice, unlike the parental HPAF-II cells that formed tumors in 5–6 days (Figure 7E). The FAXDC2-OE tumors increased in volume 1.9-fold in 3 weeks, whereas the parental HPAF-II tumors more than doubled (2.3-fold) in 1 week.

We and others have previously demonstrated that Wnt inhibition causes both differentiation and cellular senescence in Wnt-addicted cancers (14, 15, 54). Here, we tested the role of FAXDC2. As before, treatment of HPAF-II tumors with Wnt inhibitor significantly increased senescence-associated β-galactosidase (SA-β-gal) levels as well as the expression of cytokines and growth factors implicated in senescence-associated inflammation (Figure 7, F and G, and Supplemental Figure 8A). Knockout of FAXDC2 prevented this Wnt inhibition–driven senescence. Several secreted growth factors and cytokines implicated in senescence-associated inflammation, including *IL32*, *HBEGF*, *ILK*, and *CTGF*, were no longer upregulated upon Wnt inhibition in FAXDC2-KO tumors (Supplemental Figure 8A) (55–58). This suggests that the FAXDC2/RTK/MAPK pathway regulates Wnt inhibition–mediated senescence.

FAXDC2 appears to be similarly required for the differentiation seen after Wnt withdrawal. Inhibition of Wnt signaling leads to the differentiation of Wnt ligand–addicted tumors, demonstrated in part by enhanced staining for mucins and increased expression of differentiation markers such as *CDH1*, *CDHR2*, *TFF1*, and *KRT16* (Figure 7, H and I, and Supplemental Figure 8B). Notably, in the absence of FAXDC2, there was markedly diminished upregulation of these markers. Consistent with this, there was no increase in the staining for mucins in the ETC-159–treated FAXDC2-KO tumors. Thus, although tumor proliferation was partially affected by FAXDC2 knockout, the differentiation and senescence response required the increased expression of FAXDC2.

Taken together, these data show that FAXDC2 is a Wnt-repressed enzyme that regulates the abundance of specific C4-methyl sterols to facilitate cellular senescence and differentiation via hyperactivation of the RTK/MAPK signaling pathway.

### FAXDC2 regulates MAPK signaling in Wnt-driven hyperplasia of the pancreas.

We asked whether the Wnt/FAXDC2/MAPK pathway was also present in non-malignant tissues. To do this, we examined a mouse genetic model of activated Wnt-dependent signaling in the pancreas. We generated mice with homozygous deletion of *Rnf43* and *Znrf3* in the pancreas (*Ptf1a^Cre^ Rnf43^fl/fl^* *Znrf3^fl/fl^*) (59). These mice developed modest pancreatic hyperplasia (Supplemental Figure 8C) but not pancreatic cancer. As expected, they also showed an enhanced response to endogenous Wnts as evidenced by increased expression of the Wnt target gene *Tcf7* (Figure 7J). Consistent with *Faxdc2* being a Wnt-repressed gene in non-malignant tissues, there was an approximately 33% decrease in *Faxdc2* (*Gm12248*) expression in the *Ptf1a^Cre^ Rnf43^fl/fl^* *Znrf3^fl/fl^* mice (Figure 7K). Furthermore, the increase in Wnt signaling as a result of *Rnf43*/*Znrf3* deletion was accompanied by a loss of basal p-ERK staining, most prominent at the periphery of the pancreatic lobules (Figure 7L). Blocking Wnt secretion by 21 days of treatment with ETC-159 in the *Ptf1a^Cre^ Rnf43^fl/fl^* *Znrf3^fl/fl^* mice restored *Tcf7* and *Faxdc2* expression to normal levels. Importantly, this ETC-159–mediated restoration of *Faxdc2* expression was accompanied by recovery of p-ERK staining. Thus, Wnt signaling also suppresses FAXDC2 and MAPK signaling in non-malignant tissues, indicating that modulation of cholesterol biosynthesis intermediates may be a Wnt effector pathway with widespread importance.

## Discussion

Wnt signaling can regulate the balance between proliferation and differentiation in stem cells and cancers. Here, taking advantage of a potent Wnt inhibitor and a comprehensive transcriptomic analysis of a Wnt ligand–addicted orthotopic cancer model, we identified a core Wnt/β-catenin–repressed cholesterol biosynthesis enzyme, the 4-methyl sterol monooxidase FAXDC2, that controls this balance. Mechanistically, we find that inhibition of Wnt secretion led to increased abundance and activity of receptor tyrosine kinases including EGFR and EPHA2 at the cell surface and enhanced signaling via the RAS-MAPK cascade to drive widespread increases in gene expression. Upstream of these steps, we find that loss of *FAXDC2* prevents Wnt-regulated RTK activation and downstream MAPK signaling to prevent a differentiation/senescence response.

In multiple cancers, oncogenic mutations of RAS lock it in the GTP-bound “on” state, leading to activated MAPK signaling. Recent studies have demonstrated that RAS signaling is tightly regulated even in cancer, in part because excessive RAS signaling drives senescence (51–53). One unexpected finding of our recent study was that high Wnt signaling in cancers prevented hyperactivation of MAPK signaling and RAS-mediated senescence. In this context, our data suggest that one mechanism for RAS-mutant cancers to avoid excess signaling and senescence is to concurrently repress FAXDC2, thereby preventing RTK hyperactivation and inhibiting downstream MAPK signaling.

FAXDC2 is a paralog of MSMO1/SC4MOL in cholesterol biosynthesis and is required for the demethylation of the C4 position of cholesterol biosynthesis intermediates (Figure 2) (18). Not much is known about the function of FAXDC2 apart from a single report implicating it in megakaryocyte differentiation (60). FAXDC2 is both ubiquitously expressed and highly conserved through evolution in plants, animals, and sponges and contains the signature sequences present in sterol monooxidases (Supplemental Figure 2). We find that *FAXDC2* expression regulates the abundance of specific C4-methyl sterols, lophenol, and dihydro-T-MAS. These specific sterols are saturated at the 24,25 position, placing them in the Kandutsch-Russell (KR) branch of cholesterol biogenesis (19, 20). Hence, FAXDC2 may preferentially act on the cholesterol precursors in the KR arm of the cholesterol biosynthesis pathway. We note that Kandutsch and Russell initially identified 24,25-dihydrocholesterol precursors in cancer tissue, calling them tumor sterols (19, 20). Consistent with this, we find that lophenol and dihydro-T-MAS are elevated in pancreatic and colorectal cancers (Figures 2 and 3).

Lophenol is widely present in plants such as aloe vera that are ascribed medicinal properties, including activity in metabolic disorders (e.g., see ref. 61). In *Caenorhabditis elegans*, lophenol is required for dauer formation. Our data suggest a potential biological mechanism for the effects of these C4-methyl sterols in RTK/MAPK regulation, a subject we are actively investigating.

To our knowledge, FAXDC2 has not previously been identified as a Wnt-repressed gene. We were able to identify it as a Wnt/β-catenin target owing to several factors. First, our Wnt-regulated data set was obtained from in vivo, orthotopic Wnt-addicted cancer models and patient-derived xenografts, where the dynamic range of Wnt-regulated genes was much higher than that seen in subcutaneous xenografts and cultured cells (14). Second, our discovery data set contained 42 independent orthotopic tumors sequenced to high depth, giving us robust statistical power. We validated our findings in independent data sets that identified Wnt target genes using genetic rather than pharmacologic approaches. Finally, we confirmed the observational findings in independent experiments using stabilized β-catenin. Hence, our identification of FAXDC2 as a Wnt/β-catenin–repressed gene in pancreatic and colorectal cancers is made with high confidence. Determining whether FAXDC2 is a direct or indirect β-catenin target gene is an area of active investigation. This is not as straightforward as it appears, as important β-catenin/TCF4 binding sites are now well recognized to be in enhancers that are quite distant from transcription start sites (62, 63).

We and others have reported that Wnt inhibition drives differentiation of Wnt-addicted cancers. Reactivation of ephrin-Eph bidirectional signaling following Wnt inhibition could be one potential mechanism contributing to the differentiation of tumors. EPH signaling is well known to provide critical positional cues to cells during development, migration, and differentiation, including proliferation in the intestinal stem cell niche (64, 65). Future studies will test whether the FAXDC2-dependent increase in abundance of Ephs on the cell surface and consequent engagement of bidirectional signaling plays a crucial role in the differentiation response following Wnt inhibition.

MYC is a well-recognized and essential target of Wnt signaling. We previously identified 2,131 genes regulating cell cycle and ribosomal biogenesis whose transcriptional response to Wnt inhibition in vivo depended on MYC status (14). Our study shows that derepression of FAXDC2 is also a key consequence of Wnt inhibition, suggesting that FAXDC2 may be as important a Wnt target as MYC in Wnt-high cancers. Confirming the importance of the Wnt/FAXDC2 axis in regulating the senescence/differentiation response, knockout of FAXDC2 alters the expression of about 3,500 Wnt-regulated genes. Thus, activation of MYC and repression of FAXDC2 may be central to Wnt signaling for regulating the balance of proliferation versus senescence and differentiation.

To our knowledge, our study provides the first evidence for Wnt-regulated repression of the expression of FAXDC2, leading to an altered abundance of C4-methyl sterols and RTK/MAPK signaling. Future studies will address the importance of FAXDC2 and the C4-methyl sterols it regulates during development and adult tissue homeostasis.

## Methods

Further information can be found in Supplemental Methods.

### Flow cytometry.

For flow cytometric analysis, cells were seeded in 60 mm dishes and transfected with 50 μM siRNAs against *FAXDC2*, *MSMO1*, *RAB11A*, *RAB11B*, *RAB4*, *RAB25*, *RAB7*, or *EHD1* (Qiagen). Twenty-four hours after transfection, the medium was replaced with fresh medium containing DMSO or 100 nM ETC-159. After 72 hours of treatment, non-permeabilized cells were incubated with EGFR-FITC (SC-120, Santa Cruz Biotechnology), ERBB2-PE (98710, Cell Signaling Technology), EphA2 (ab150304, Abcam), or EphB2-APC (564699, BD Biosciences) antibodies for 60 minutes. Goat anti-rabbit IgG (H+L) Alexa Fluor 594 secondary antibody (A-11012, Thermo Fisher Scientific) was used for EphA2. Cells were washed with PBS and cell staining assessed on a BD Fortessa. The data were analyzed using FlowJo software v10.0.7.

### Colocalization assays.

Epitope-tagged FAXDC2, MSMO1, and NSDHL cDNA was cloned into a pcDNA3.1 mammalian expression vector using restriction enzymes. All tags were located at the N-terminus. FAXDC2 and MSMO1 were tagged with V5. NSDHL was tagged with FLAG. Seventy-five thousand HeLa cells were plated on coverslips in 12-well plates and transiently transfected with the relevant plasmids (MSMO1 and NSDHL or FAXDC2 and NSDHL) using Lipofectamine 2000 (Invitrogen) (10 ng of plasmid per well). Forty-eight hours after transfection, cells were fixed in 4% paraformaldehyde and permeabilized using a commercially available saponin-based permeabilization and blocking reagent (1× BD Perm/Wash buffer, 554723, BD Biosciences). Cells were then stained using primary antibodies against FLAG (20543-1-AP, Proteintech; 1:2,000) and V5 (MCA1360, Bio-Rad; 1:500) and subsequently with fluorophore-labeled anti-mouse (goat anti-mouse Alexa Fluor 488, Invitrogen; 1:2,000) and anti-rabbit secondary antibodies (goat anti-rabbit Alexa Fluor 594, Invitrogen; 1:2,000). Duolink Mounting Medium with DAPI was used to mount the coverslips. Images were obtained using a Nikon Ti2-E microscope with a ×100 oil immersion objective and were analyzed using NIS-Elements software.

### Generation of MSMO- and FAXDC2-KO cells.

To generate MSMO- or FAXDC2-KO HPAF-II cells, MSMO1 sgRNA (sg: 5′-CACCGCAGAGACATGGGAAAACCAA-3′) or FAXDC2 sgRNAs (sg3: 5′-TCTTGTTCTACTATTCACAC-3′; sg4: 5′-TGGGGAAAGATATCATGCAC-3′) were cloned into FgH1tUTCyan (85551, Addgene) and FgH1tUTG (70183, Addgene) plasmids, respectively.

Lentiviral particles were produced by transient transfection of 293T cells grown in 60 mm Petri dishes with 10 μg of vector DNA along with the packaging constructs pMD2.G (12259, Addgene) and psPAX2 (12260, Addgene) using Lipofectamine 2000. Virus-containing supernatants were collected at 48–72 hours after transfection and passed through a 0.45 mm filter. HPAF-II cells stably expressing FUCas9Cherry (70182, Addgene) were transduced with virus-containing supernatant with 10 ng/mL Polybrene. Genome editing was validated by PCR amplification and sequencing.

### Low-density assays.

HPAF-II cells (9,000 cells per well) were plated in 24-well cell culture plates for low-density assays. HPAF-II cell pools with inducible single guide (isg) RNAs against FAXDC2 or MSMO1 or FAXDC2-KO cells with isgRNAs against MSMO1 (FAXDC-KO isgMSMO) were plated in growth medium with delipidated FBS (900123, Gemini Bio). The cells were treated with 2.5 μg/mL doxycycline or DMSO and were allowed to grow for approximately 10 days. The cells were then stained with 0.5% crystal violet solution (V5265, Sigma-Aldrich), and the images were acquired on GelCount (Oxford Optronix). Crystal violet dye was solubilized by incubation with 0.1% SDS for 4–5 hours at room temperature, and the absorbance was measured at 570 nM using a Bio-Rad xMark Microplate Spectrophotometer.

### Cholesterol assay.

To measure total cholesterol, 400,00 cells per well were plated in 6-well cell plates in growth medium with delipidated FBS and harvested after treatment with either 2 μg/mL of doxycycline or DMSO. To extract lipids, 1 million cells were resuspended in 200 μL of chloroform/isopropanol/NP-40 (7:11:0.1, vol/vol) followed by centrifugation at 15,000*g* for 10 minutes at room temperature. After solvent extraction, the samples were dried in a Speedvac at 50°C for 30 minutes to remove residual traces of organic solvent. The dried lipid extracts were dissolved in 200 μL assay diluent, and total cholesterol was measured using Amplex Red Cholesterol Assay Kit (catalog A12216, Invitrogen) per the manufacturer’s protocol.

### Statistics.

Data were analyzed using Prism v5.0 (GraphPad) and R/Bioconductor. Significance for all tests was set at *P* ≤ 0.05 unless otherwise stated. Statistical tests included Mann-Whitney *U* test and unpaired 2-tailed *t* test.

### Study approval.

The animal studies were approved by the Duke-NUS Institutional Animal Care and Use Committee and complied with applicable regulations. Institutional review boards of SingHealth (2018-2795) approved the analysis of colorectal cancer samples.

### Data availability.

The RNA-Seq data were deposited in the NCBI’s Sequence Read Archive under accession PRJNA549884. RNA-Seq analysis code is available at https://github.com/harmstonlab/wnt_faxdc2_manuscript/releases/tag/v1.0.0 (commit ID b18a997cc09960b773f210eee9082b7e9952e6ec).

## Author contributions

BM and DMV designed the study. BM, SRW, and SP performed animal studies and biochemical assays. NH performed the bioinformatics analysis. WDN performed sterol structure identification and bioinformatics of FAXDC2 versus MSMSO1/SMO1 and contributed extensively to biosynthesis reasoning. ET and IBHT provided the human colorectal cancer tissue samples. EP, BM, and DMV supervised the study. BM, NH, and DMV wrote the manuscript with significant input from WDN. All authors read and approved the manuscript.

## Supplementary Material

Supplemental data

Unedited blot and gel images

Supplemental table 1

Supplemental table 2

Supplemental table 3

Supplemental table 4

Supporting data values

## Version 1 01/23/2024

In-Press Preview

## Version 2 03/15/2024

Electronic publication
