# Supplementary material for: The cholesterol biosynthesis enzyme FAXDC2 couples Wnt/β-catenin to RTK/MAPK signaling
Source: J Clin Invest. 2024 Jan 23;134(6):e171222. doi: 10.1172/JCI171222 (PMC10940096; doi:10.1172/JCI171222)

Full unedited gel for Figures 1 D and E

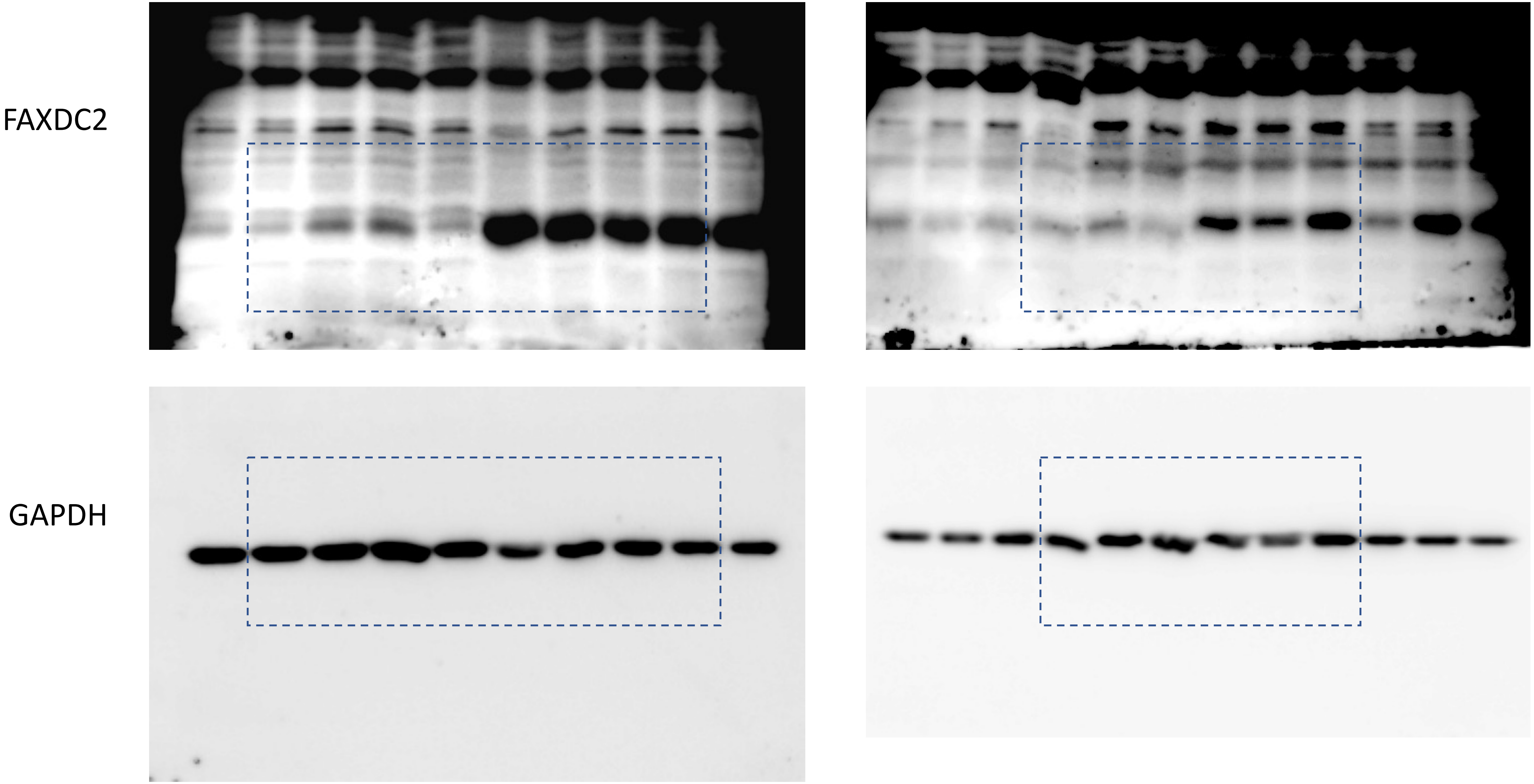

Full unedited gel for Figure 4F

pTyr

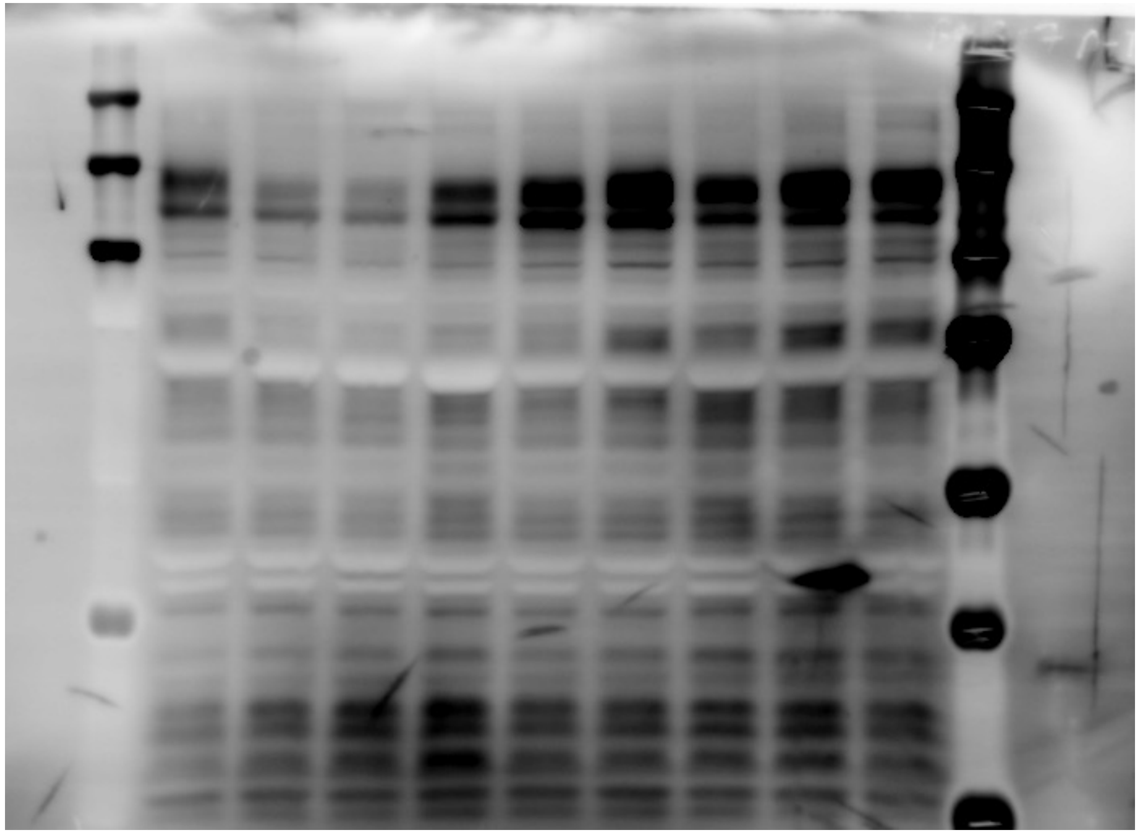

ACTIN

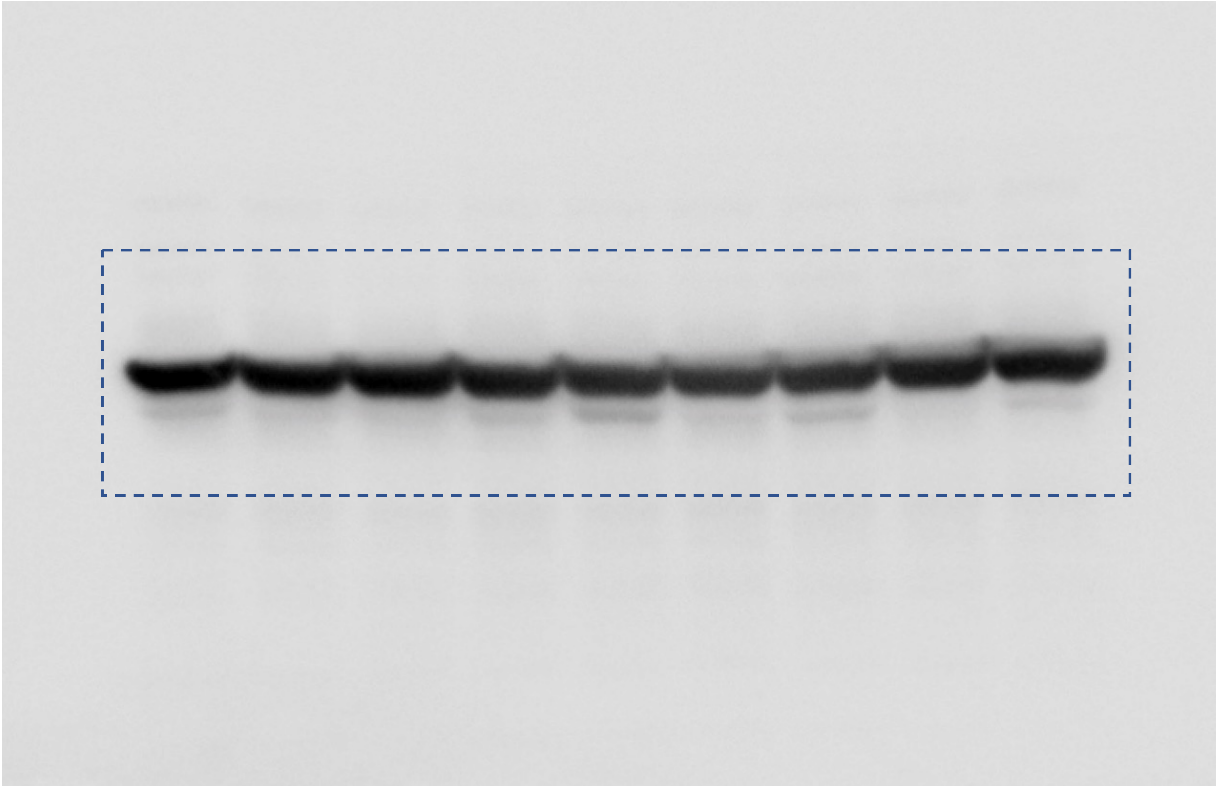

Full unedited gel for Figure 4G

pTyr

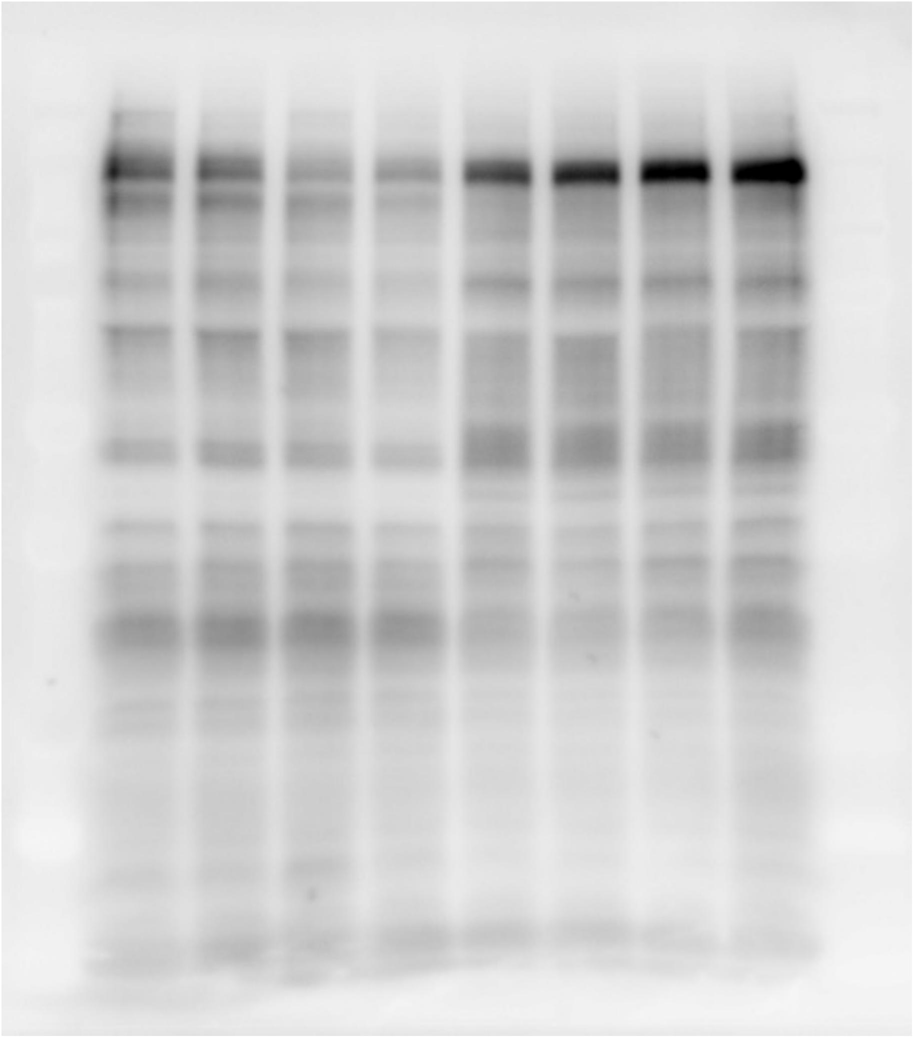

GAPDH

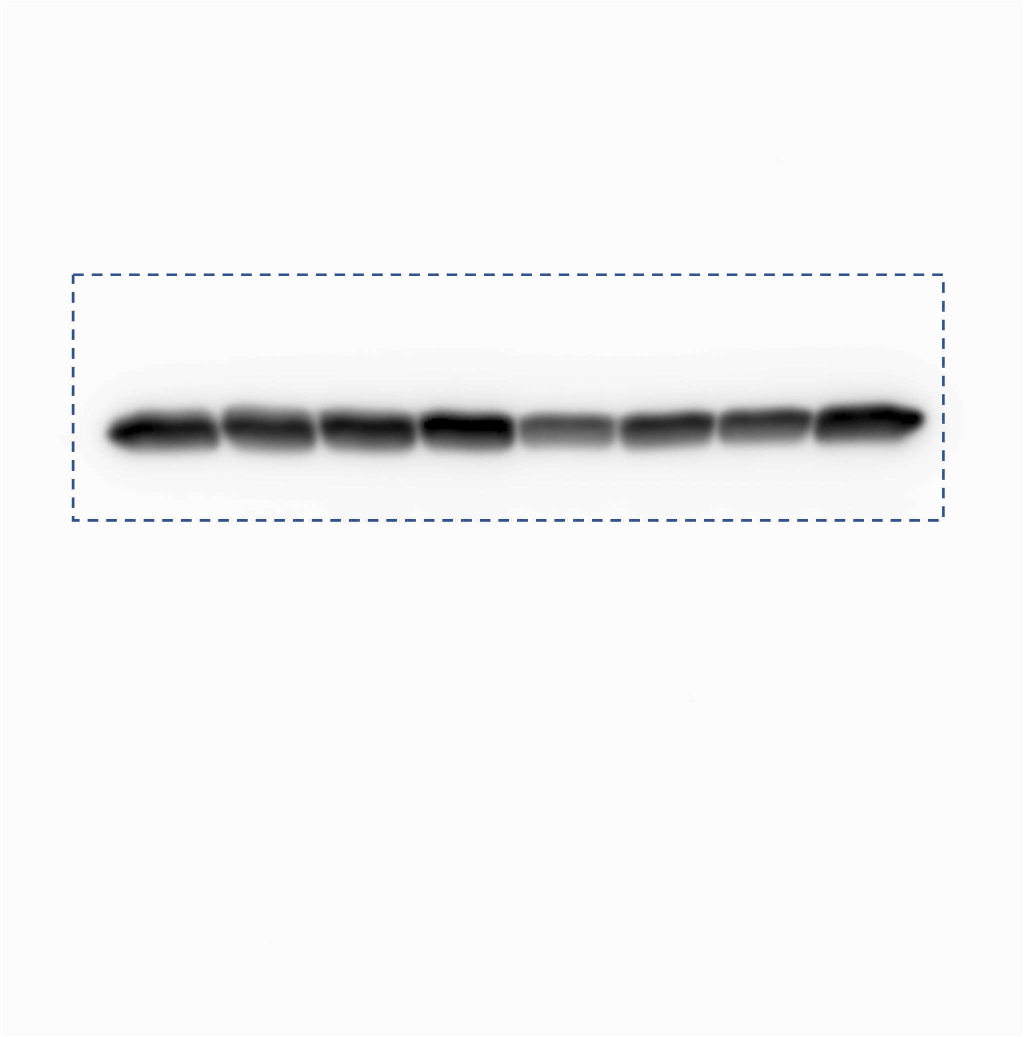

Full unedited gel for Figure 4H

pTyr

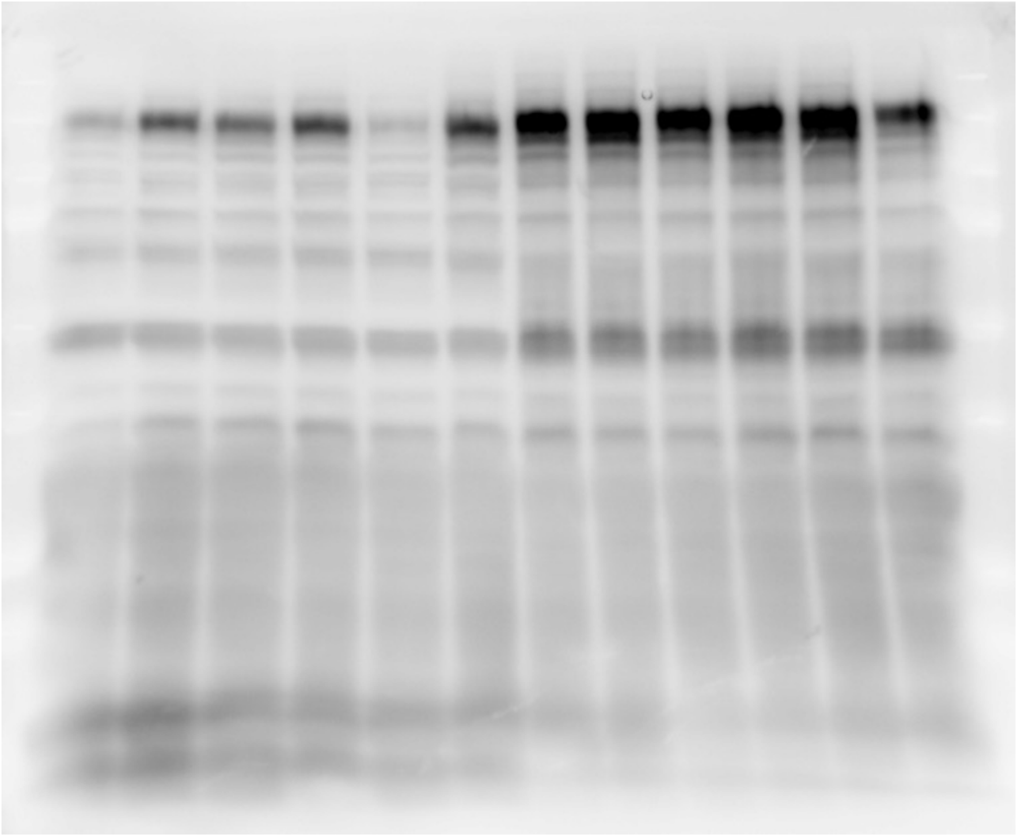

GAPDH

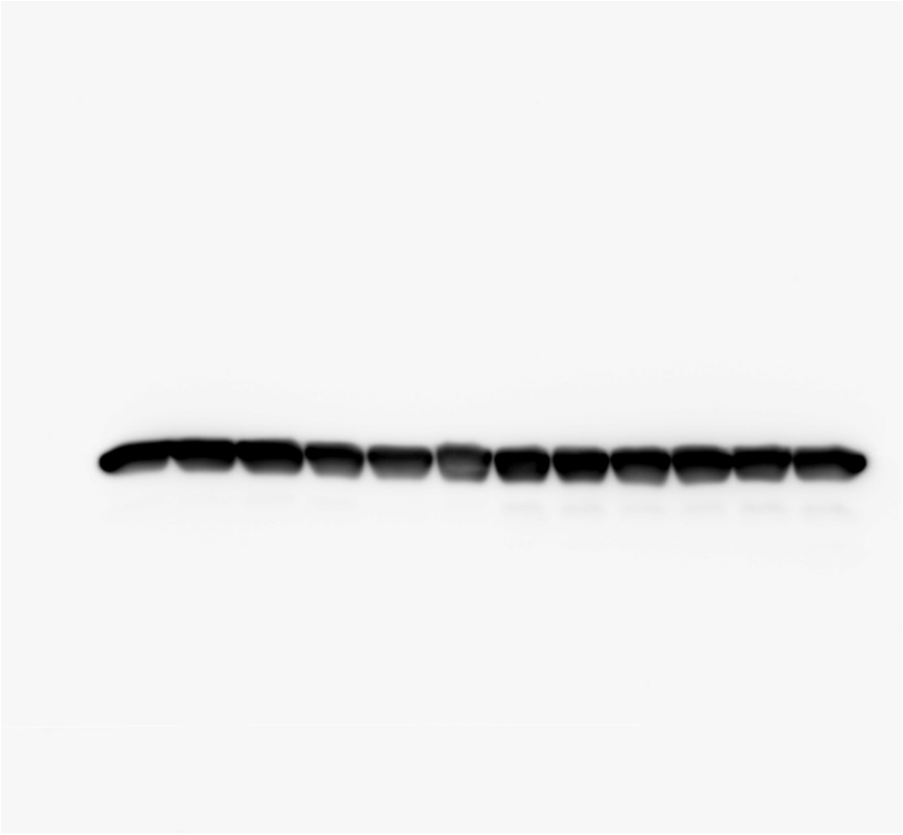

Full unedited gel for Figure 5F

pEphA2

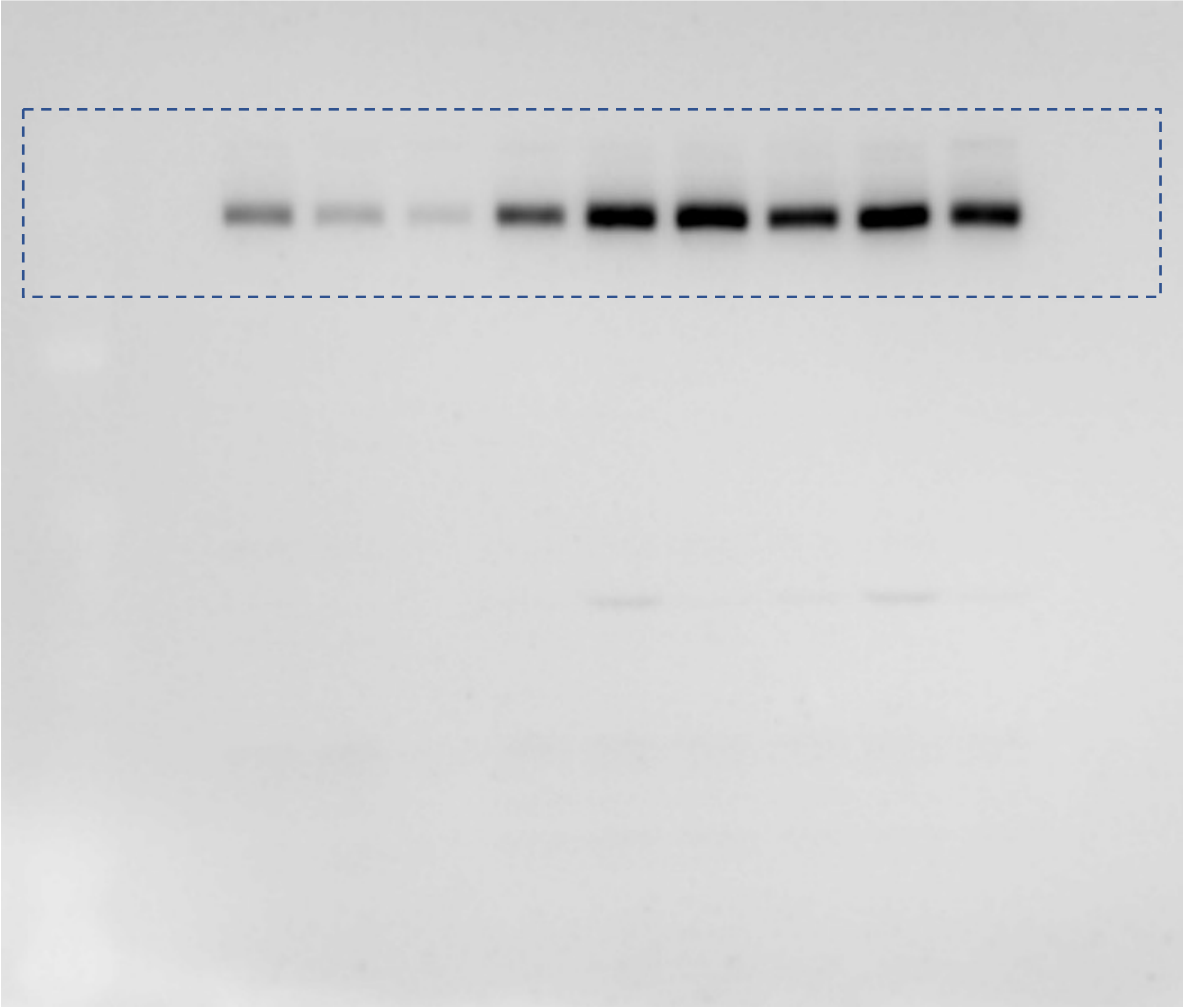

GAPDH

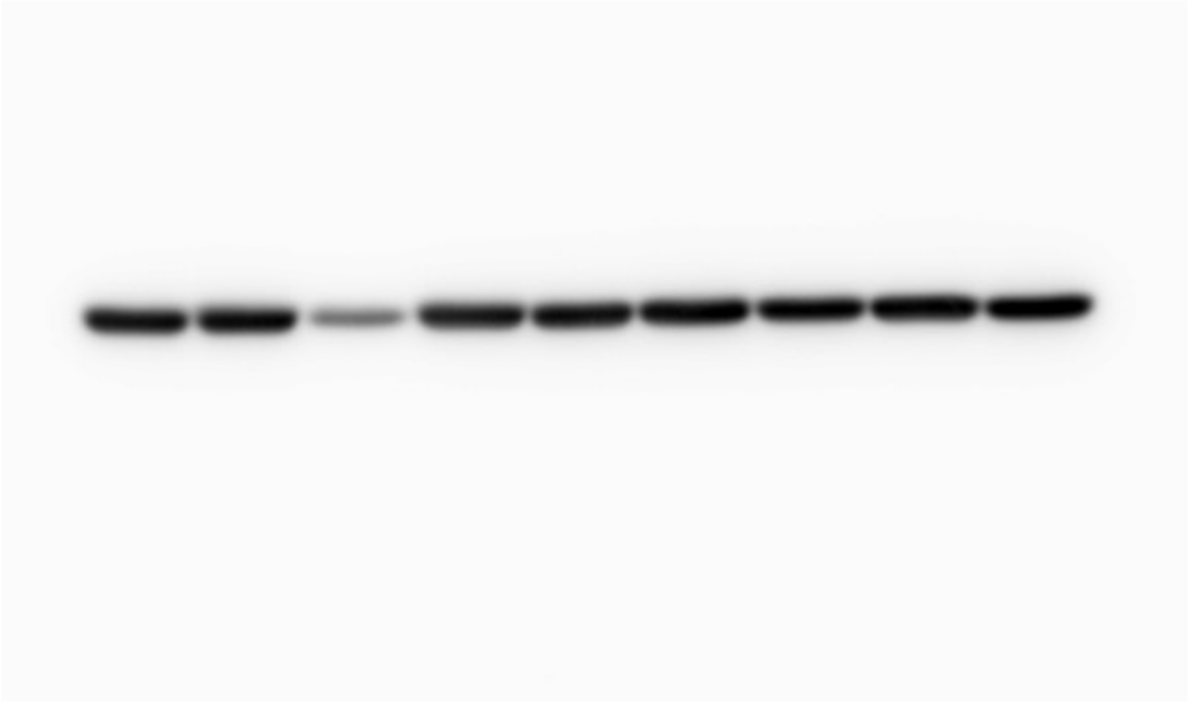

Full unedited gel for Figure 5F

pEGFR

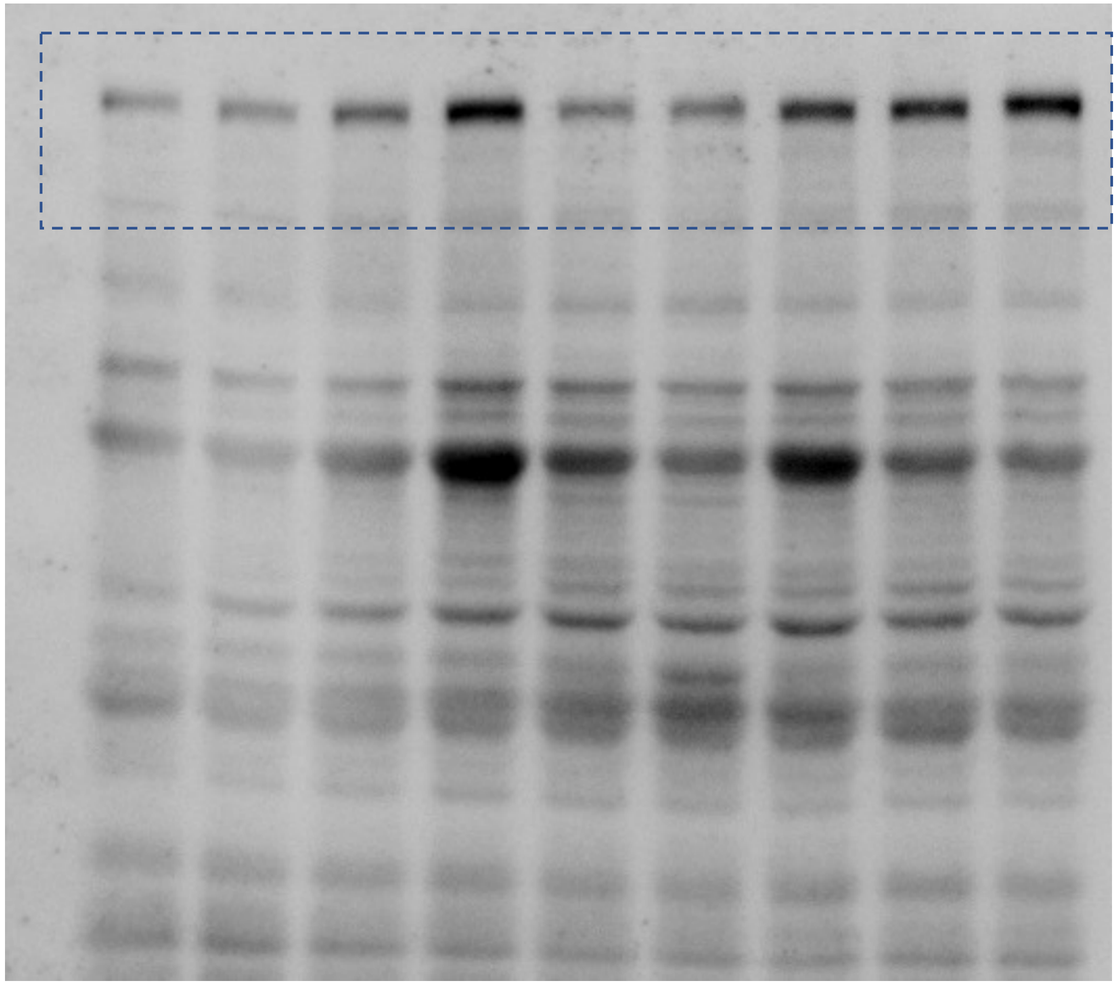

ACTIN

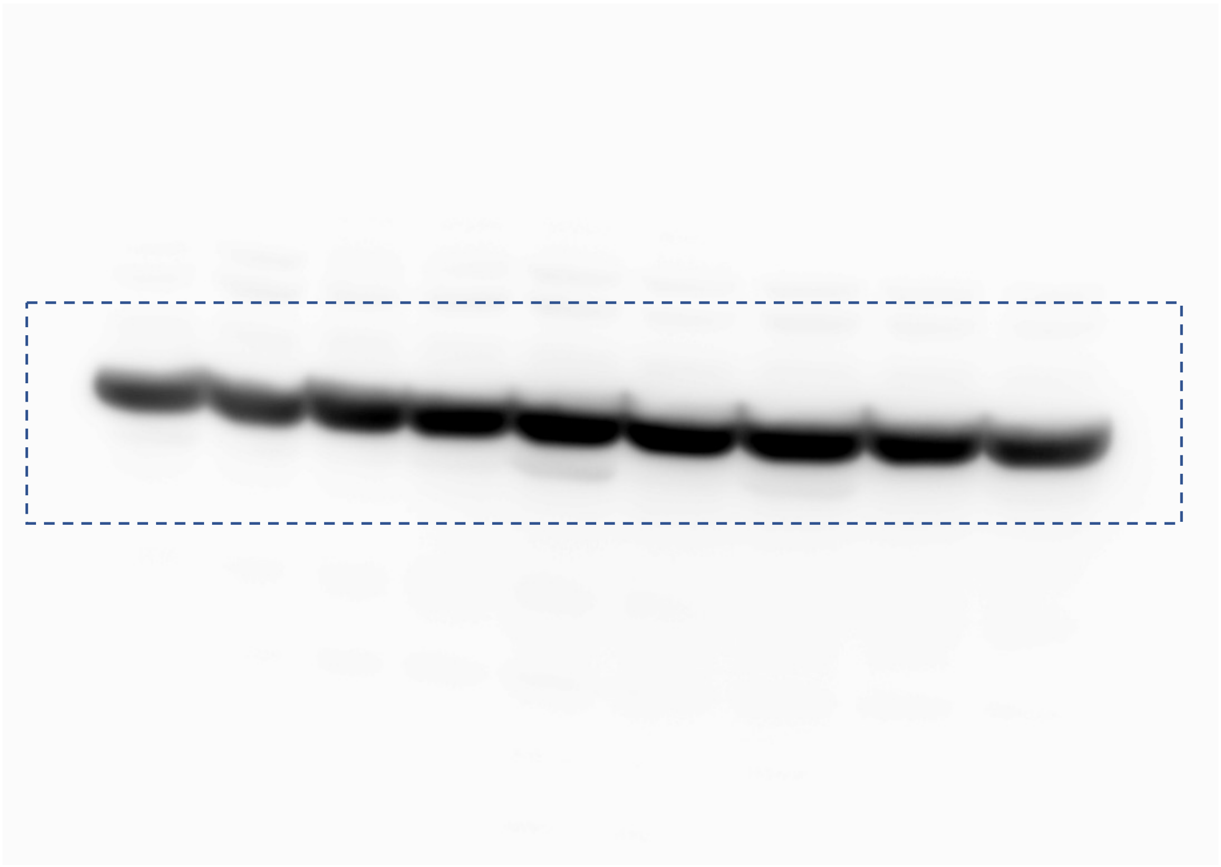

Full unedited gel for Figure 5G

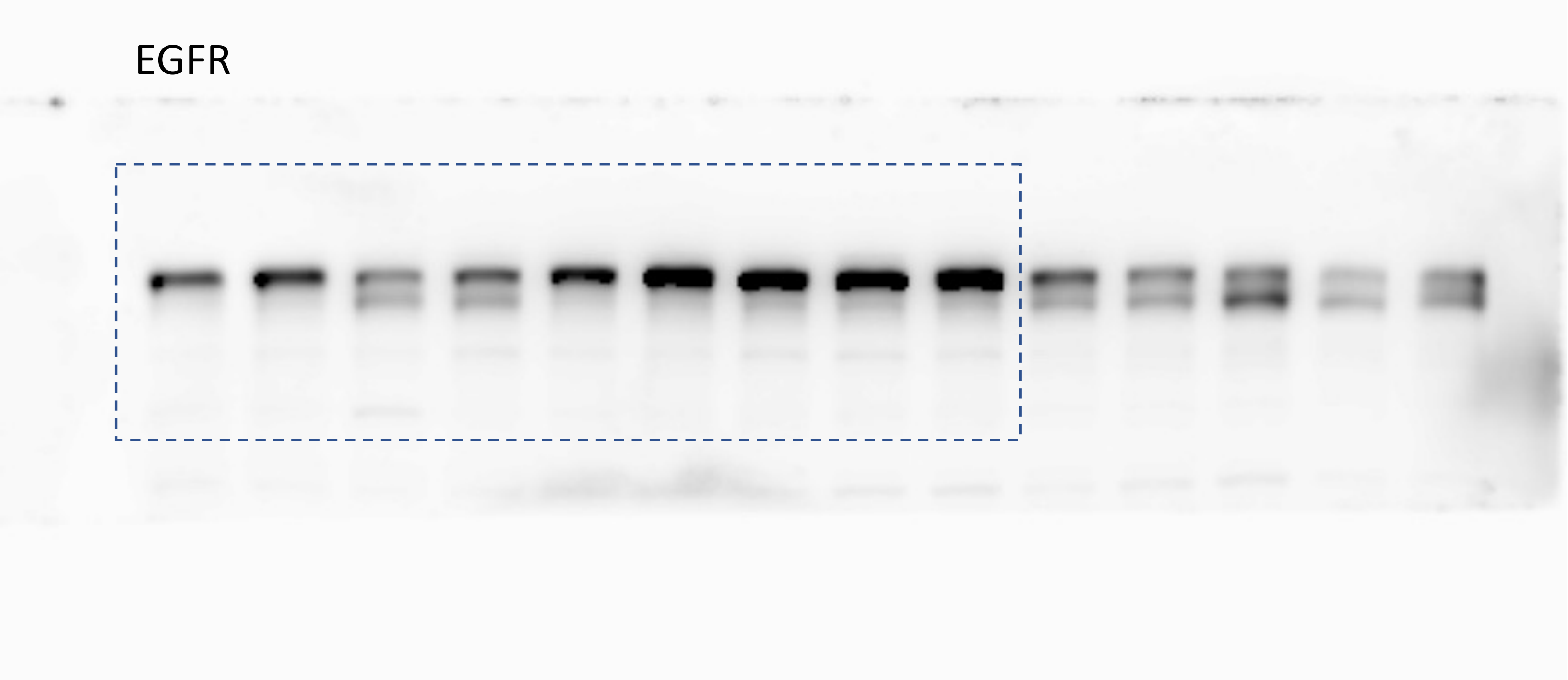

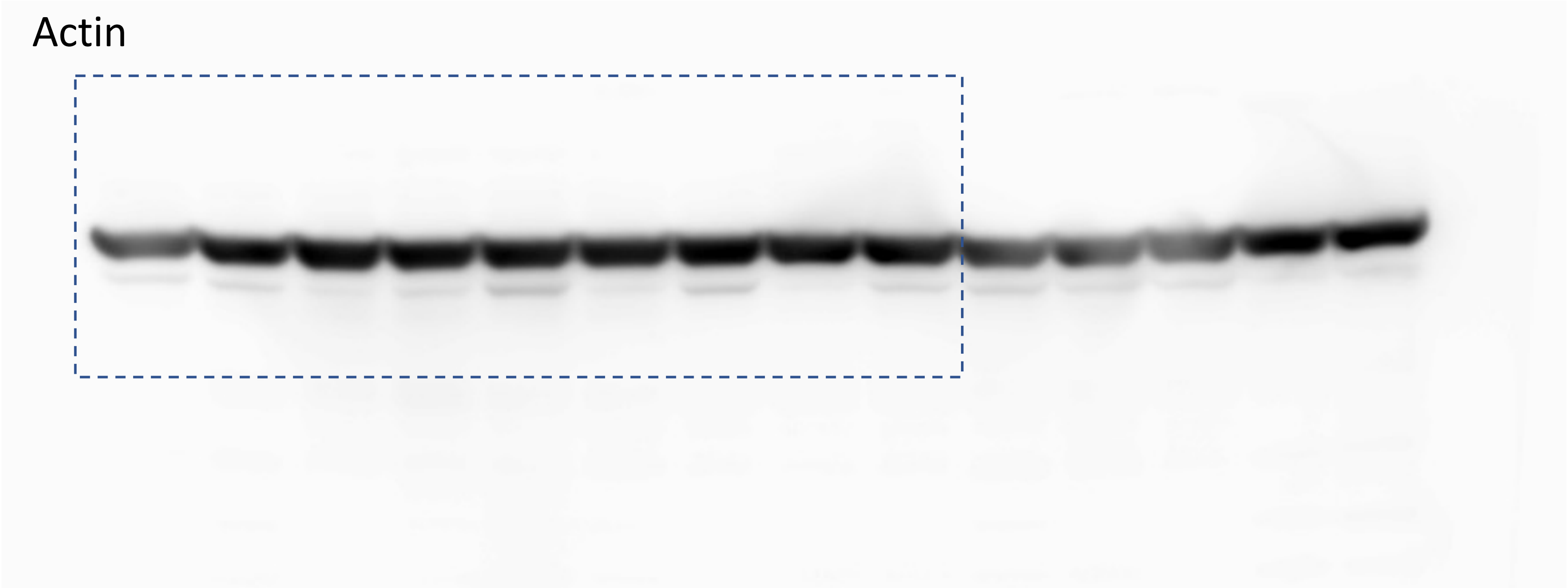

Full unedited gel for Figure 5G

ErbB2

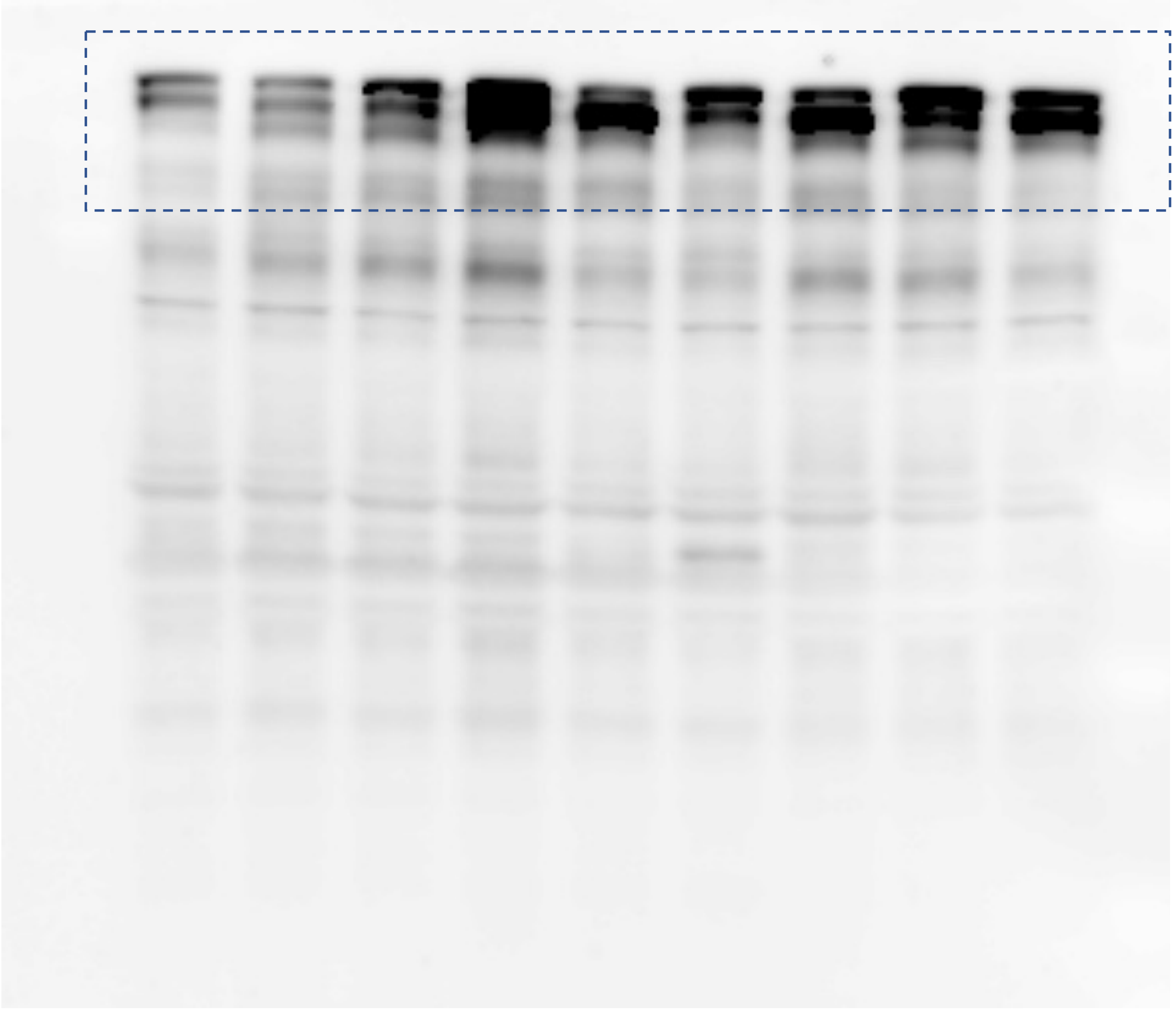

Actin

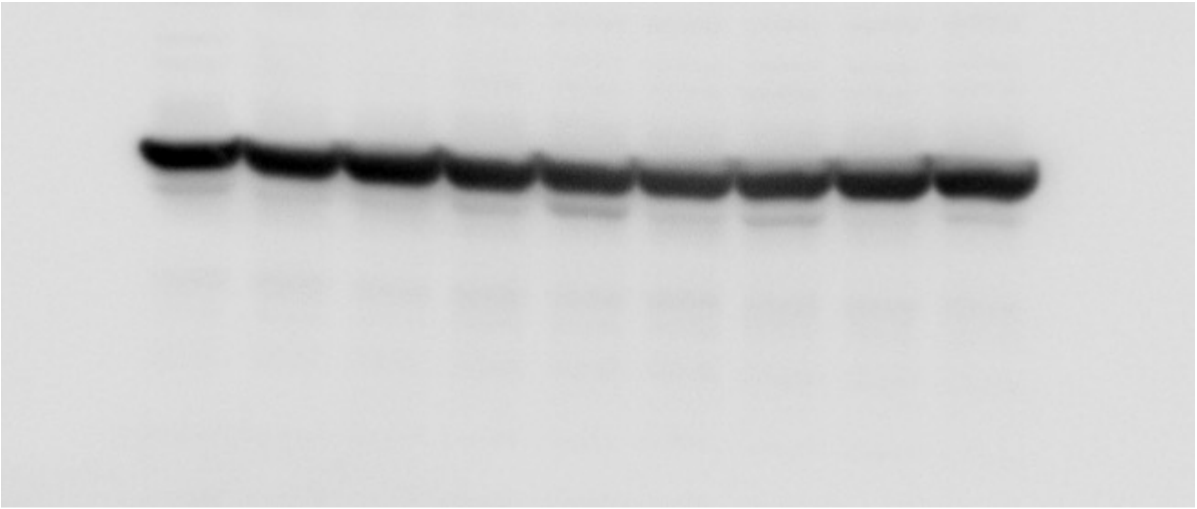

Full unedited gel for Figure 5H

EphA2

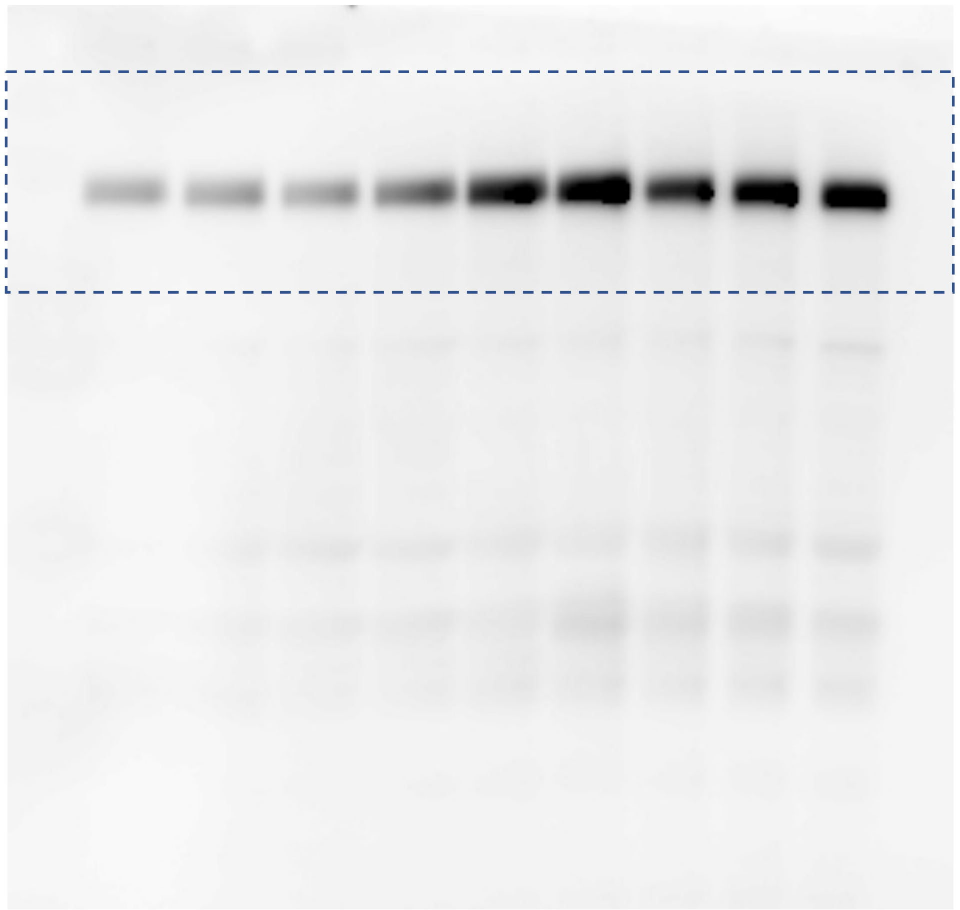

EphB2

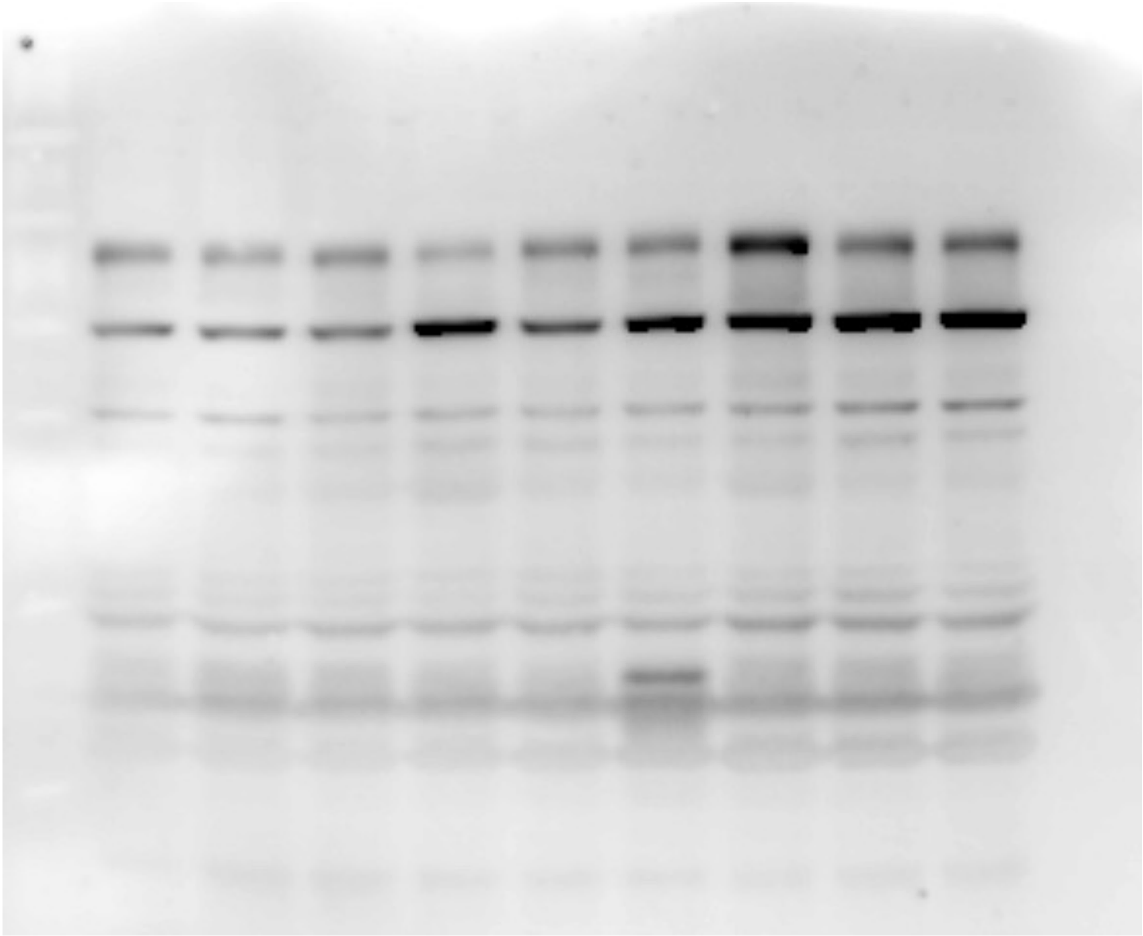

Full unedited gel for Figure 5H

EphB4

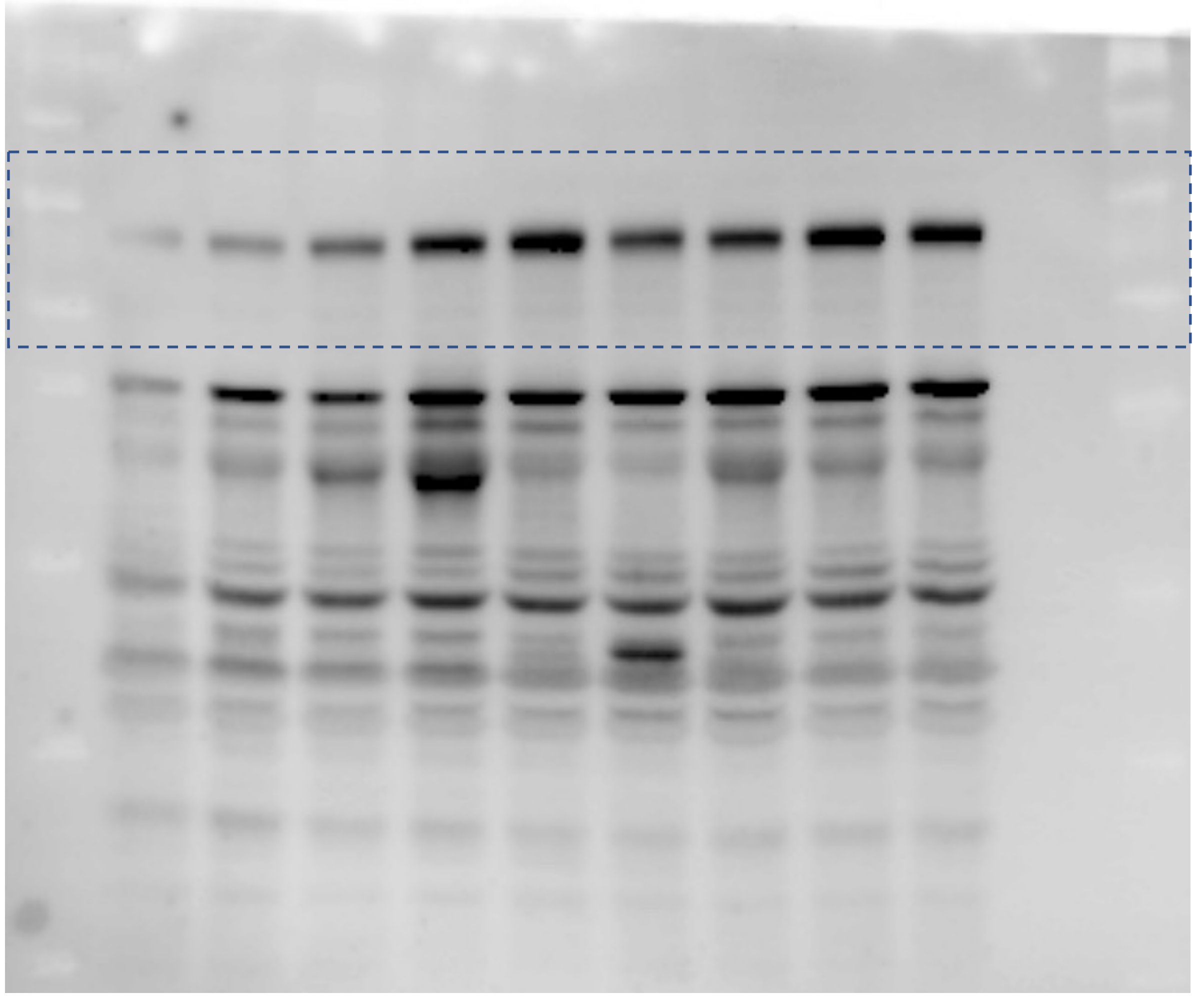

Actin

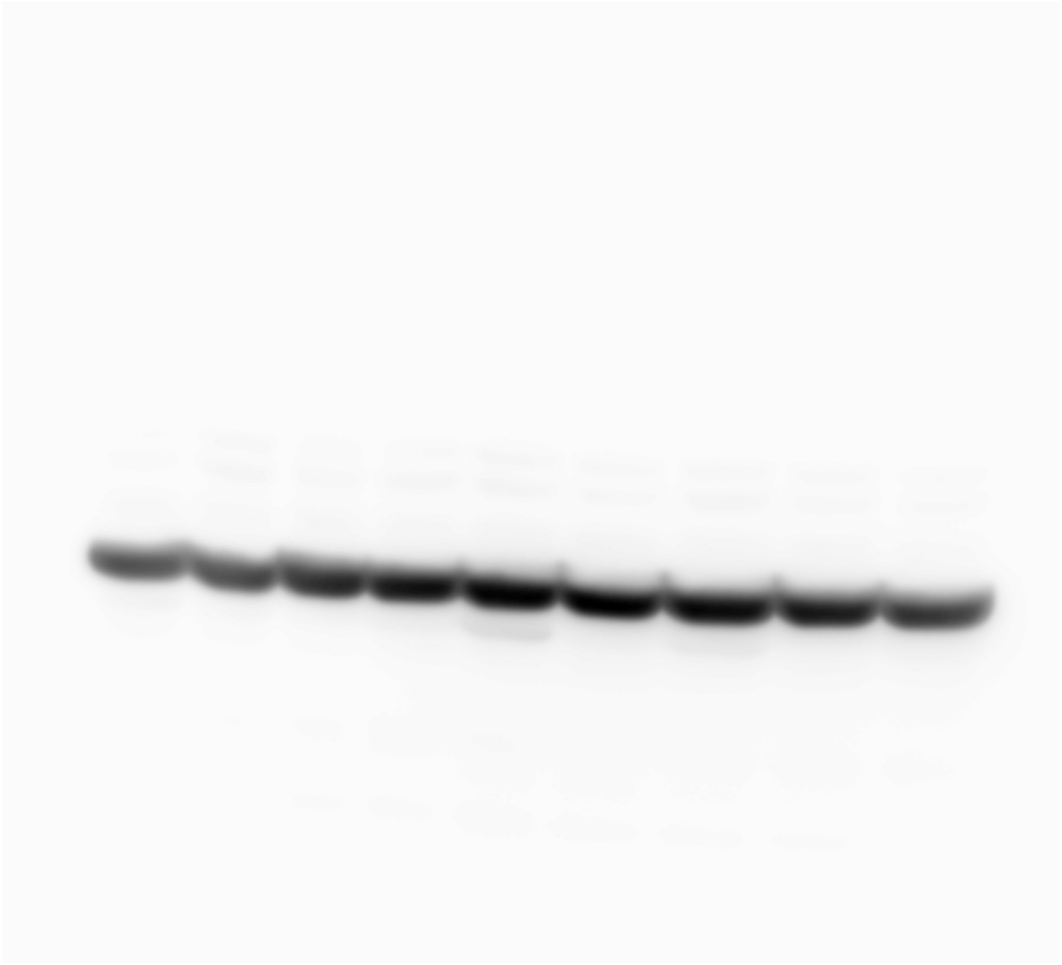

4 gels were run using equal volume from the same master mix and one of the gels was probed for actin (pEGFR, EphA2, EphB2, EphB4)

Full unedited gel for Figure 5I

EphA2

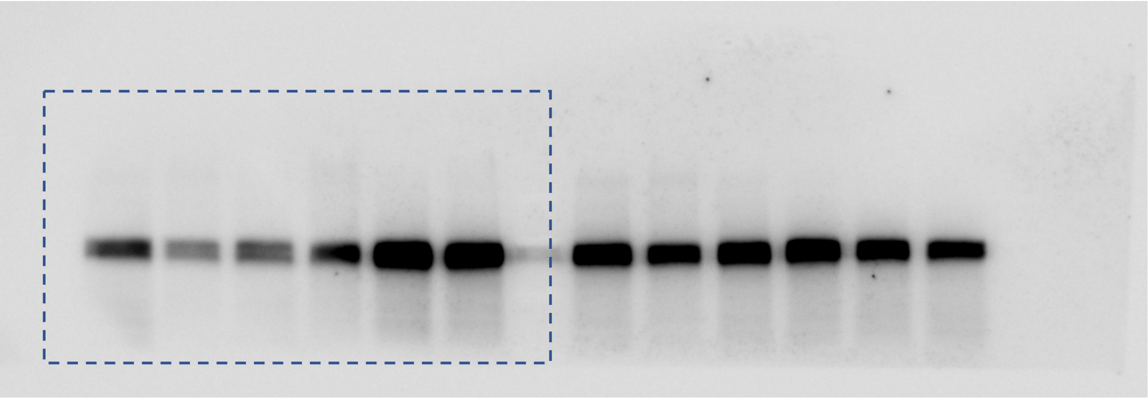

EphB4

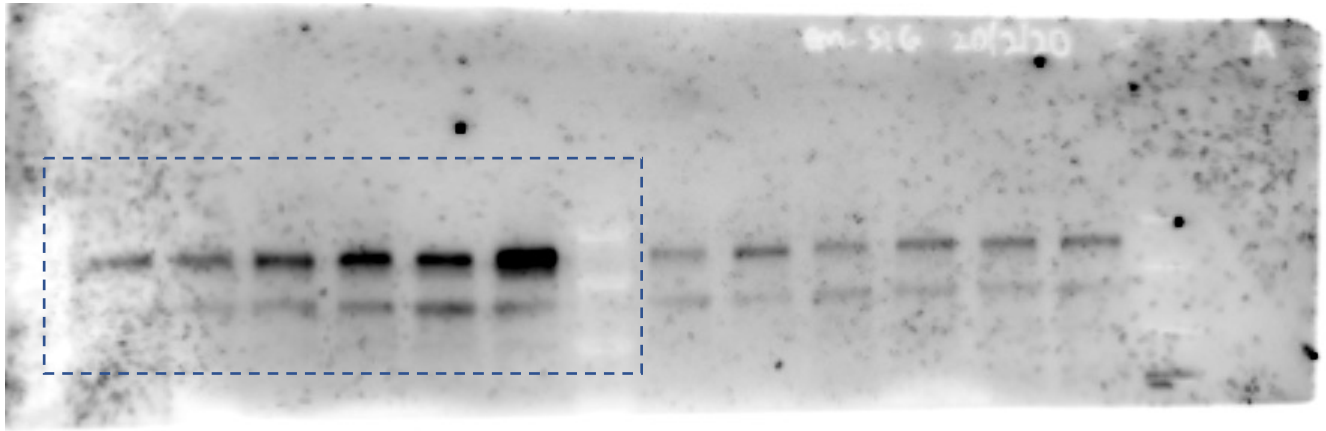

EGFR

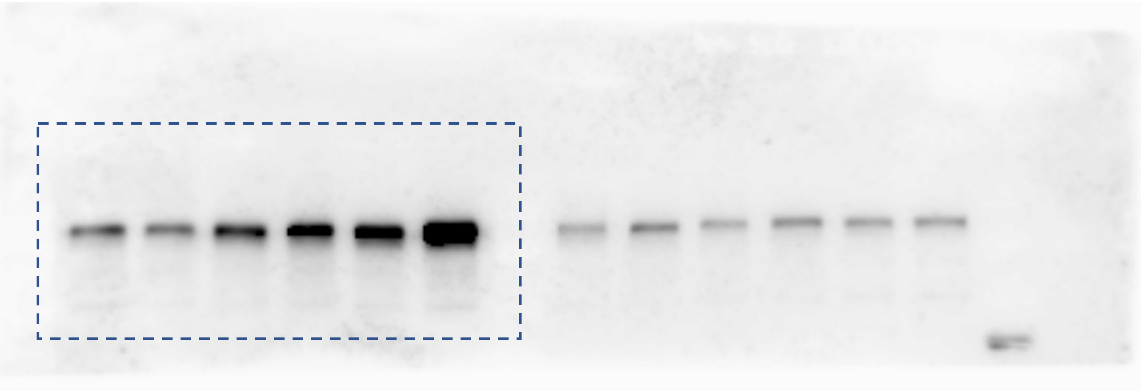

GAPDH

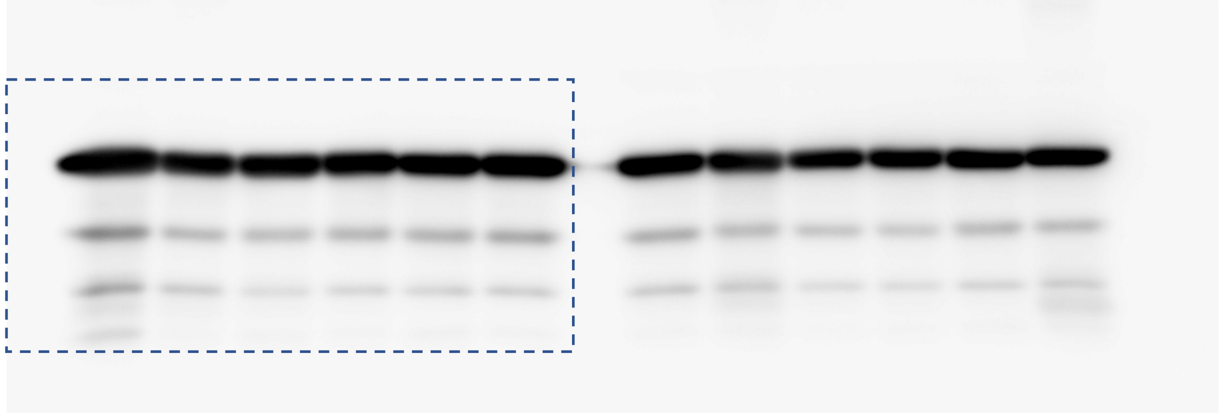

Full unedited gel for Figure 6C

pTyr

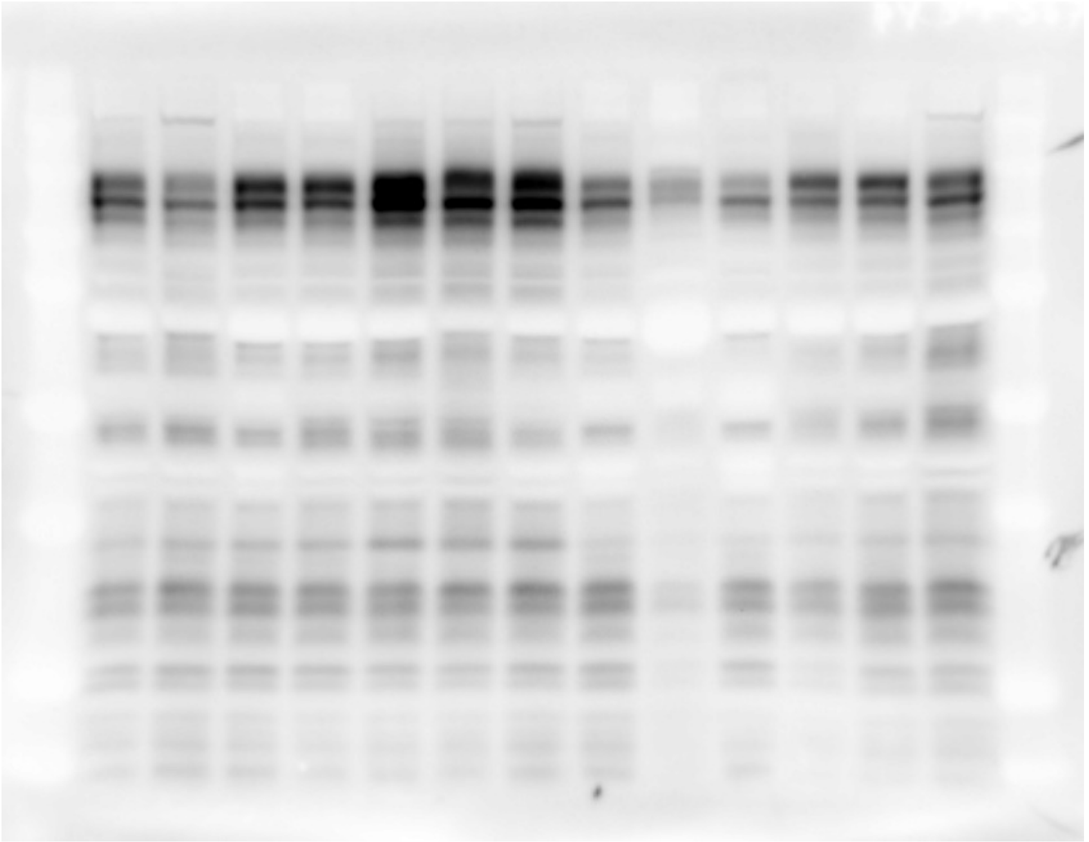

GAPDH

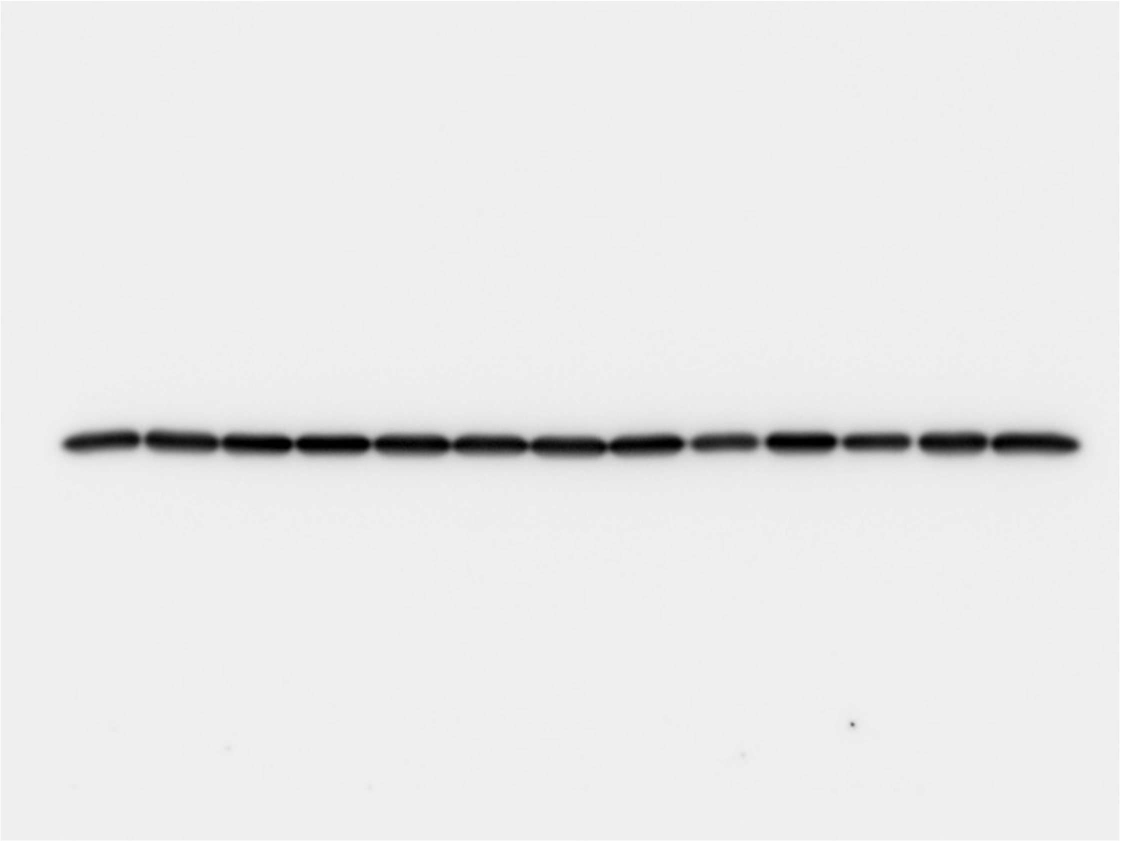

Full unedited gel for Figure 6D

pEphA2

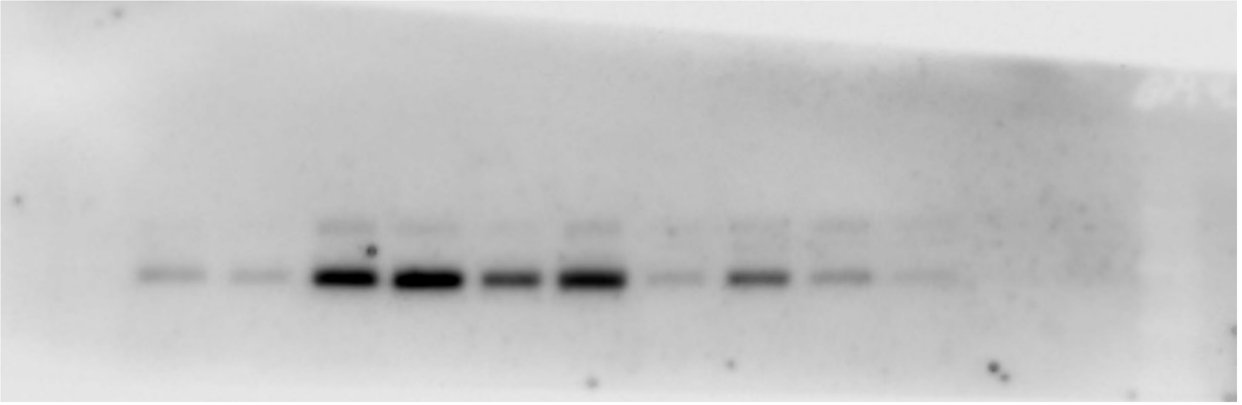

GAPDH

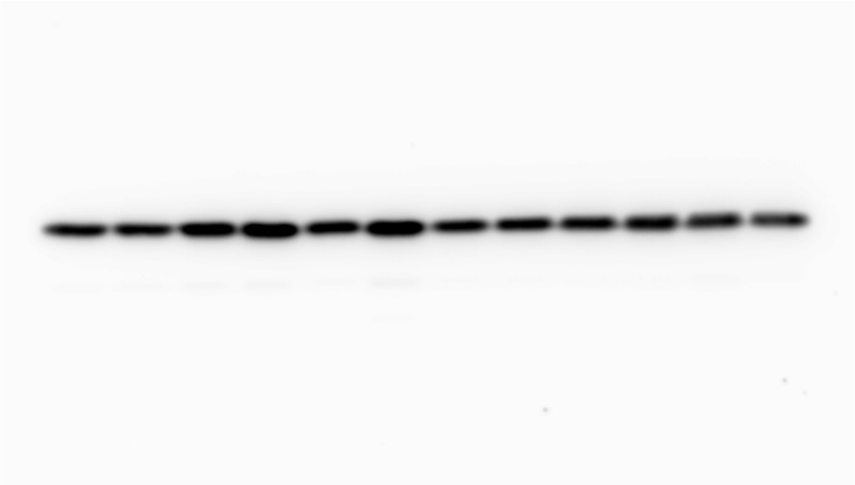

TEphA2

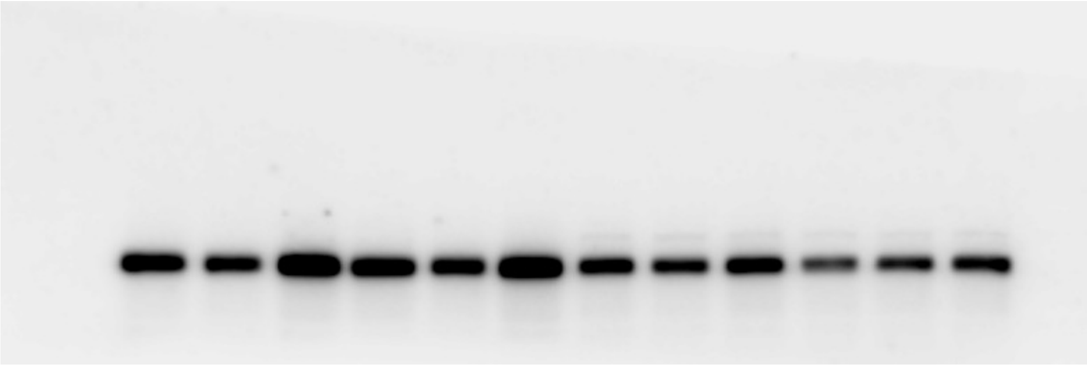

Full unedited gel for Figure 6E

pTyr

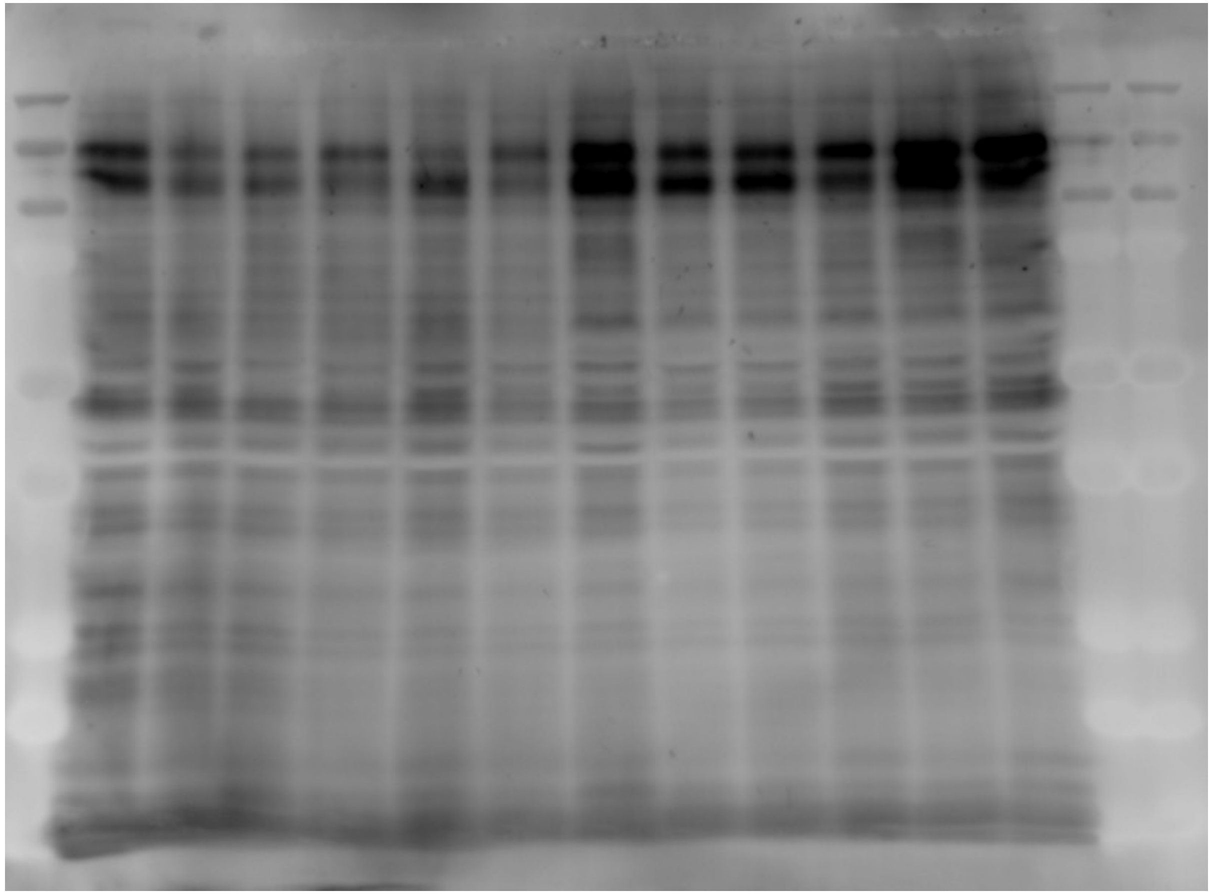

GAPDH

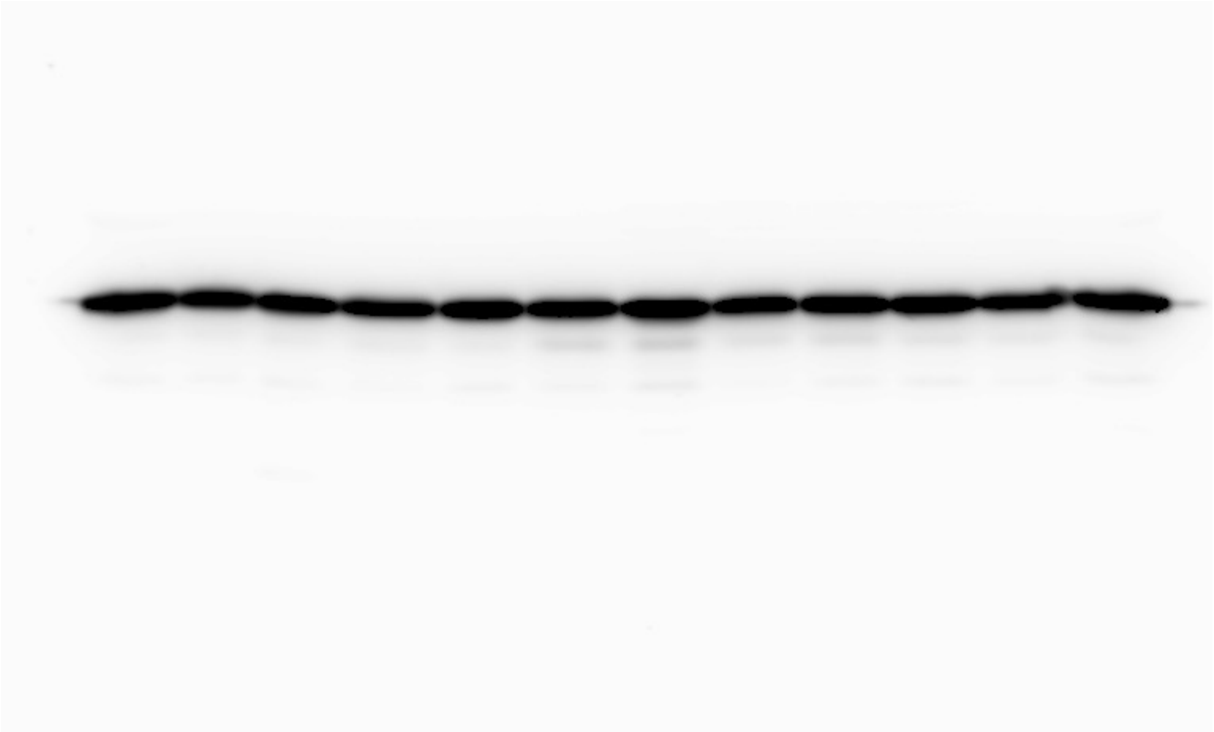

Full unedited gel for Figure 6F

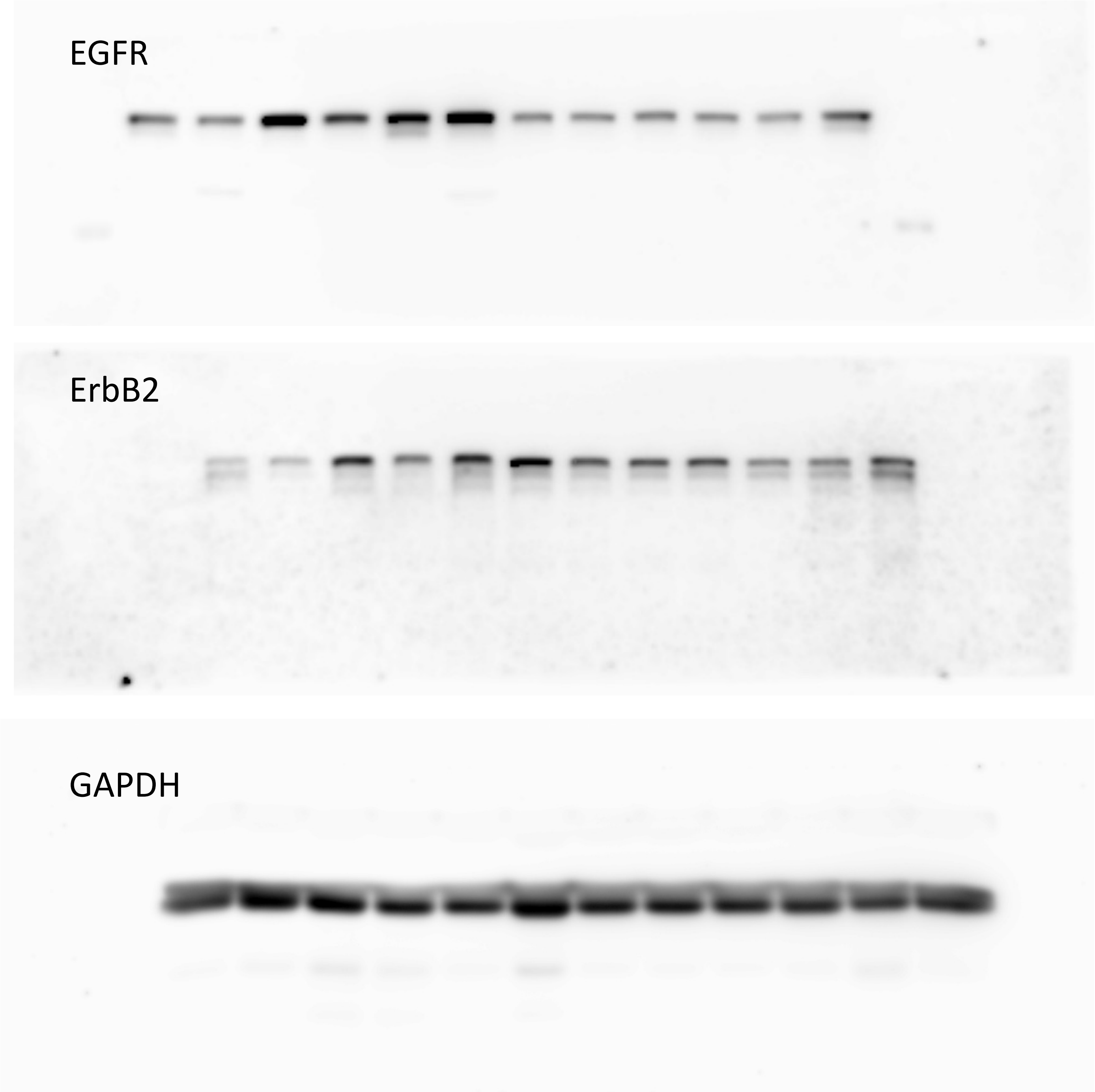

Full unedited gel for Figure 6F

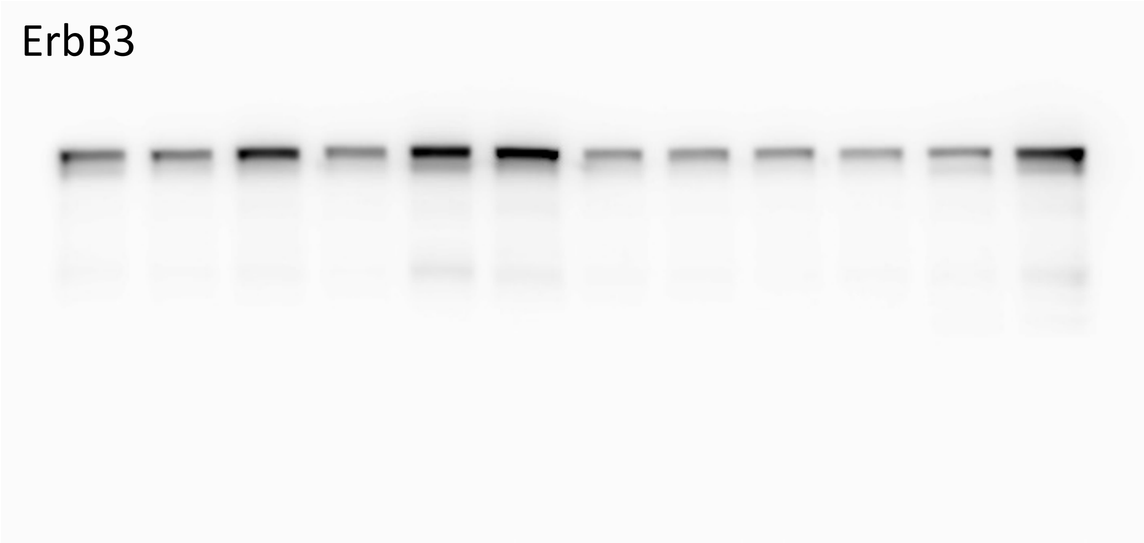

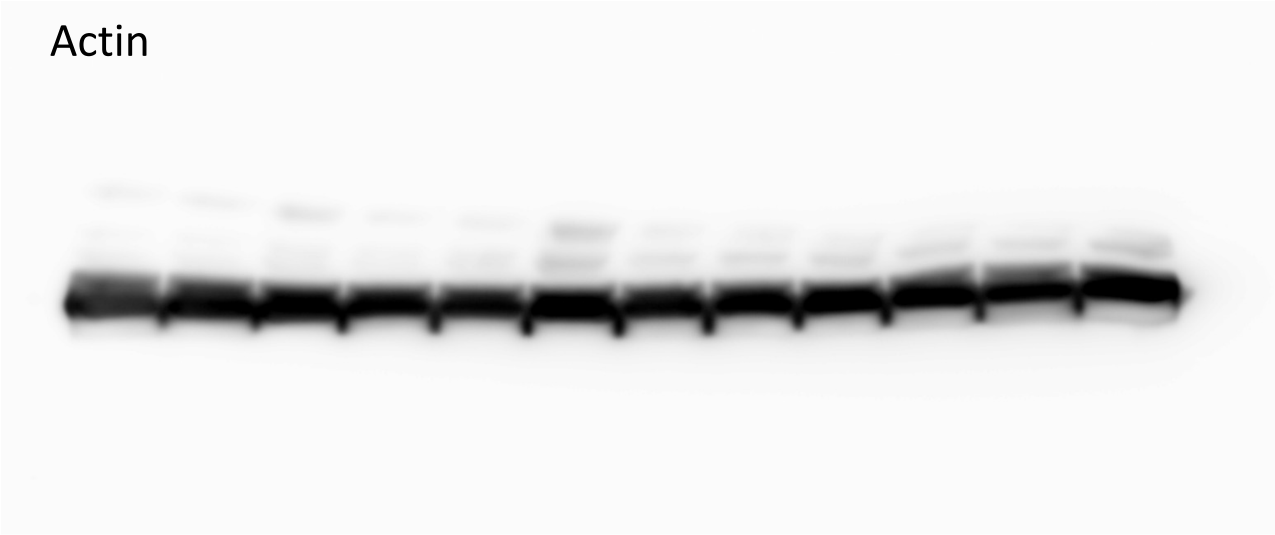

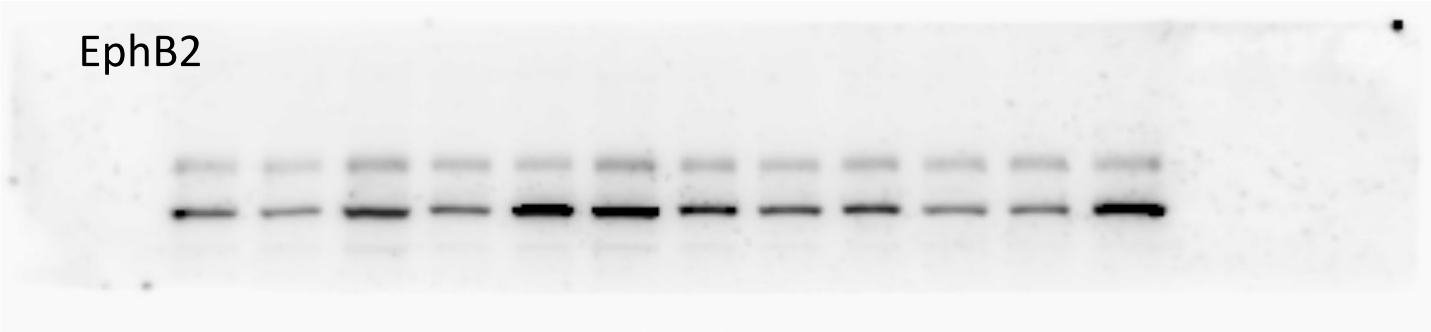

Full unedited gel for Figure 6F

EGFR

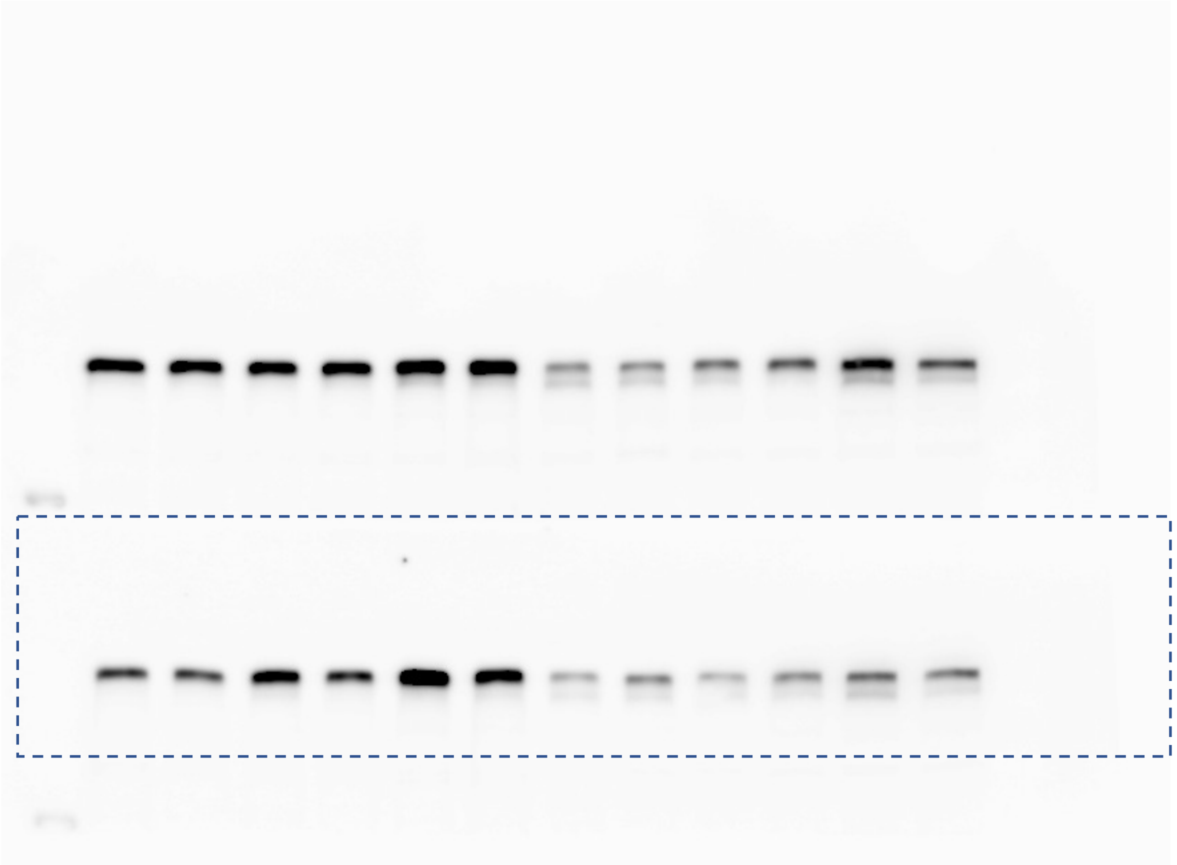

GAPDH

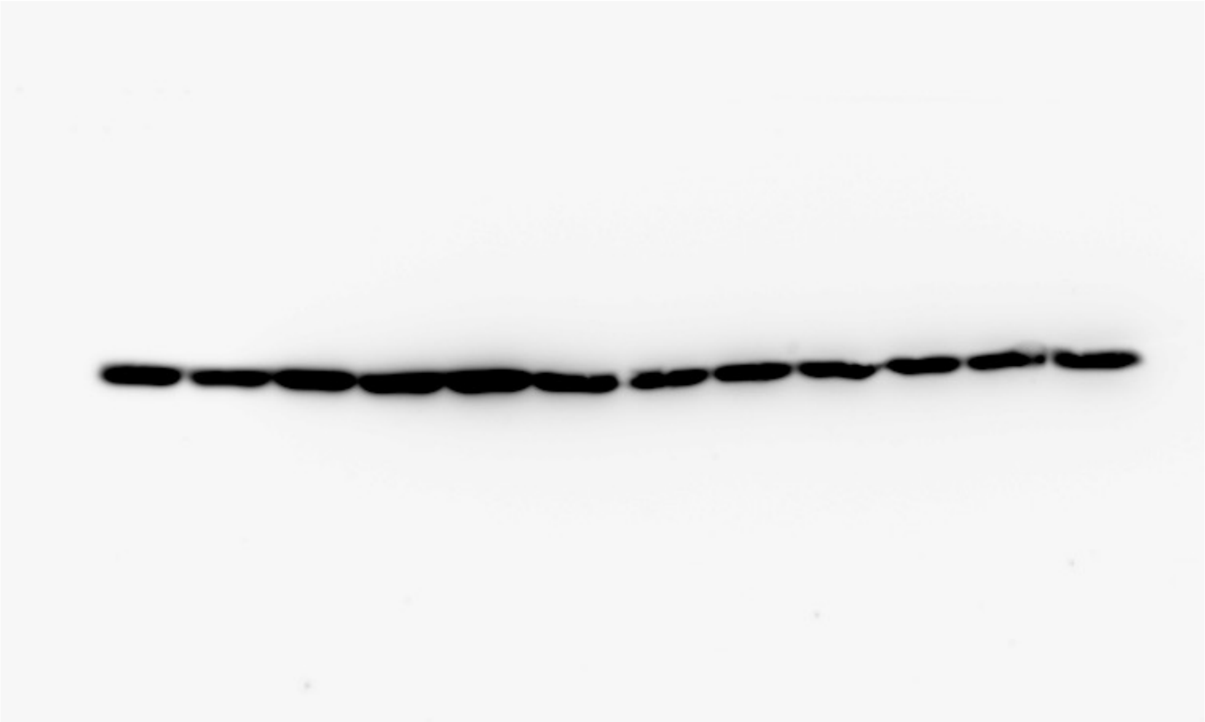

These are 2 independent blots developed at the same time. Top half is another gel

Full unedited gel for Figure 7A

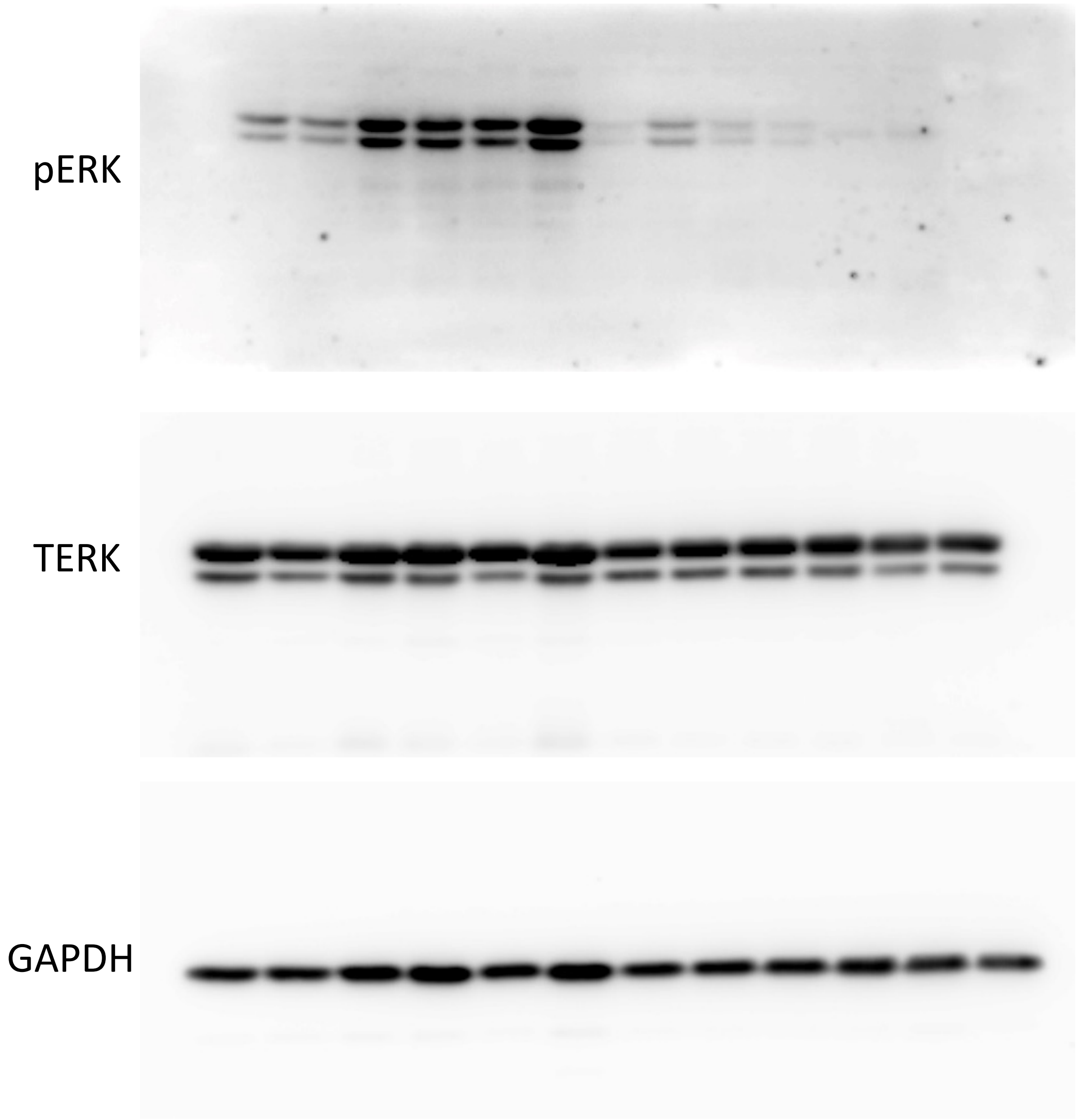

Full unedited gel for Figure 7B

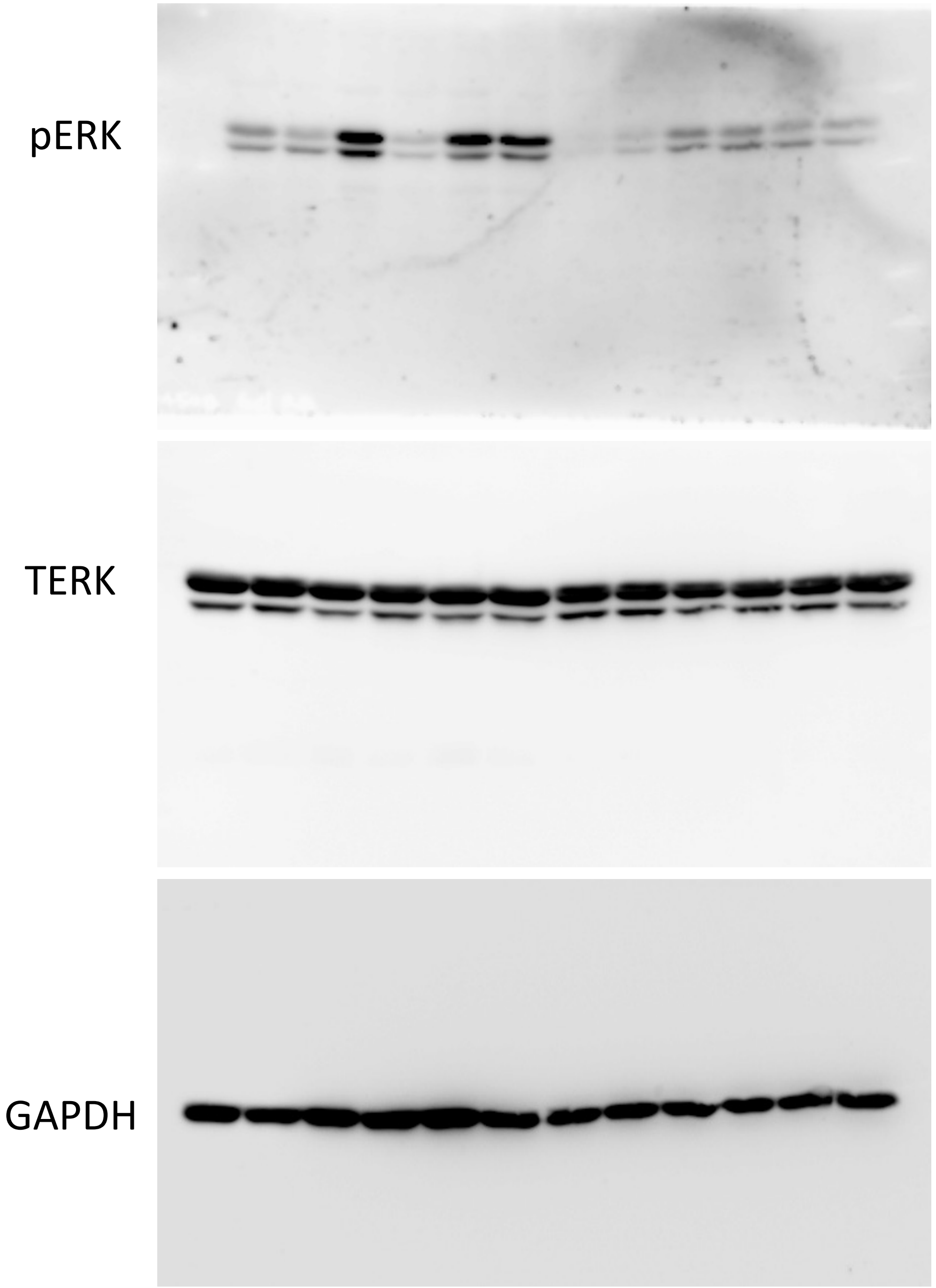

Full unedited gel for Figure 7C

pERK

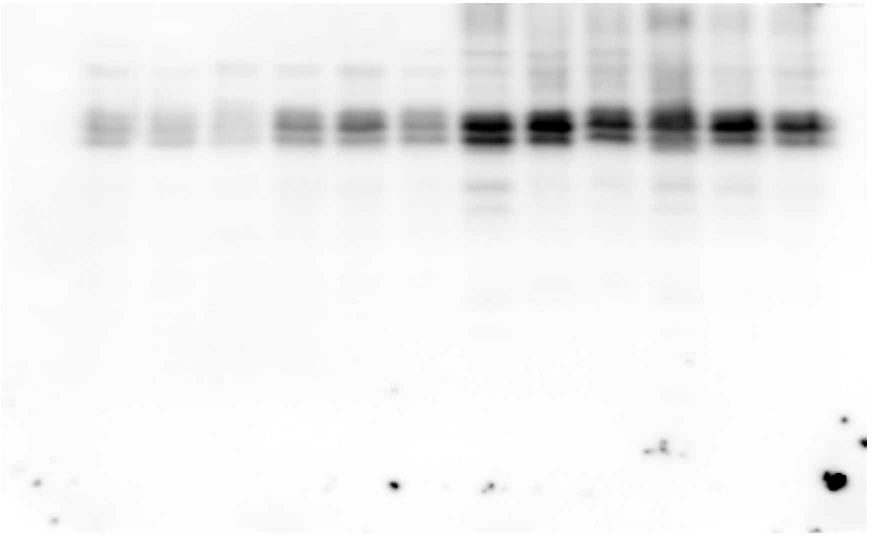

GAPDH

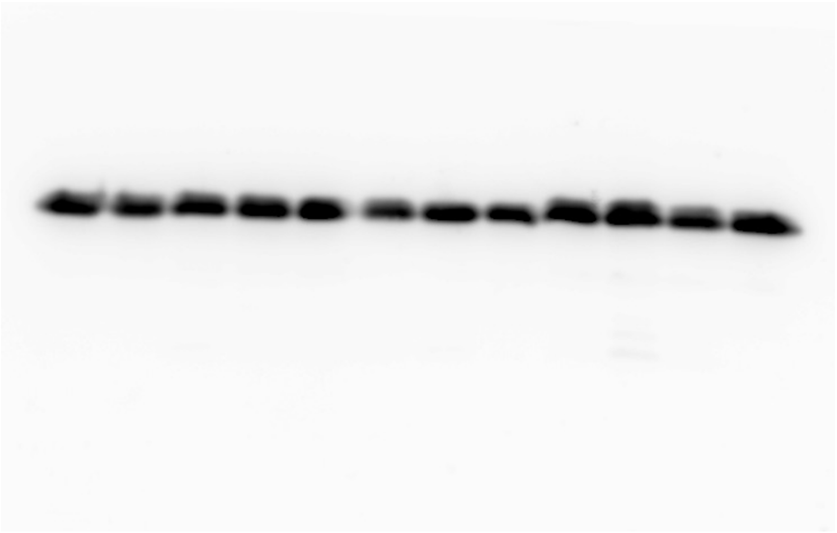

TERK

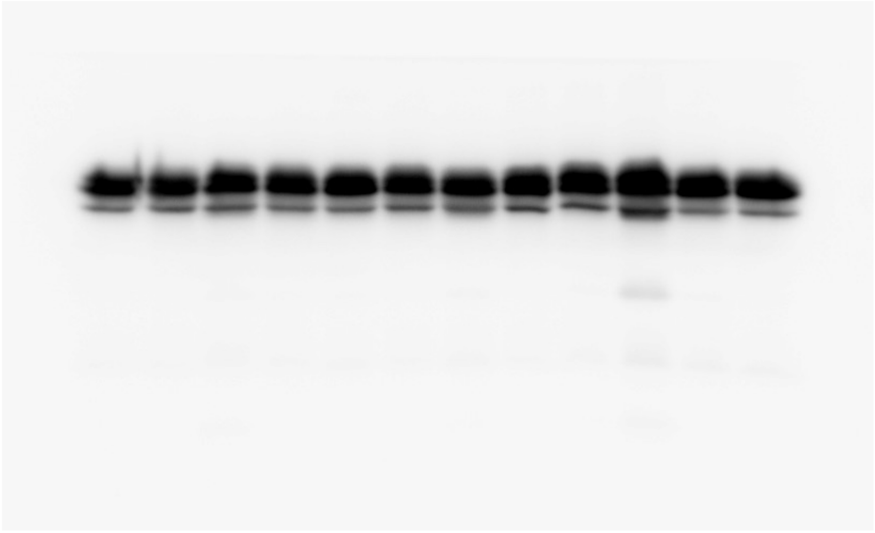

Full unedited gel for Figure S4D

FAXDC2

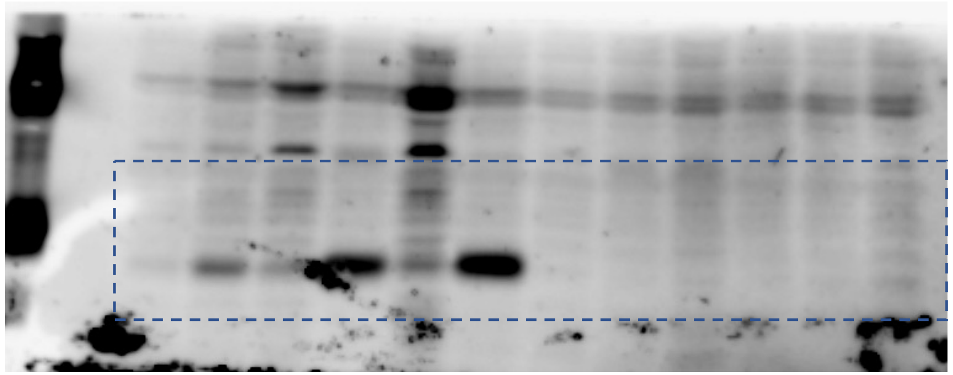

GAPDH

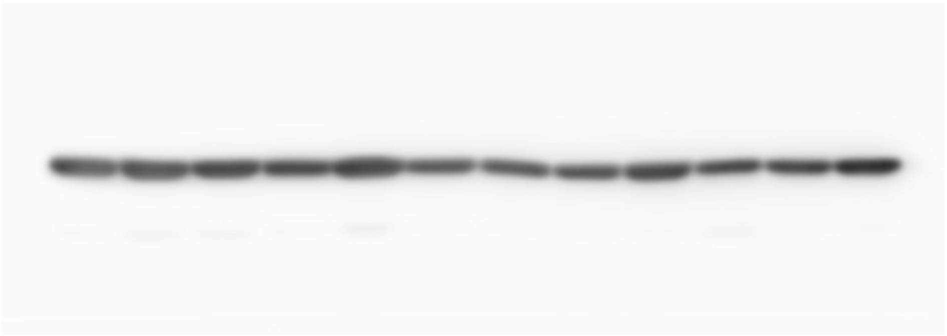

Supplement: Unedited blot and gel images [file jci-134-171222-s085.pdf]
